# Systematic analysis, comparison, and integration of disease based human genetic association data and mouse genetic phenotypic information

**DOI:** 10.1186/1755-8794-3-1

**Published:** 2010-01-21

**Authors:** Yonqing Zhang, Supriyo De, John R Garner, Kirstin Smith, S Alex Wang, Kevin G Becker

**Affiliations:** 1 Gene Expression and Genomics Unit, National Institute on Aging, National Institutes of Health, Baltimore, MD 21224 USA; 2 Division of Computational Bioscience, Center for Information Technology, National Institutes of Health, Bethesda, MD 20892 USA

## Abstract

**Background:**

The genetic contributions to human common disorders and mouse genetic models of disease are complex and often overlapping. In common human diseases, unlike classical Mendelian disorders, genetic factors generally have small effect sizes, are multifactorial, and are highly pleiotropic. Likewise, mouse genetic models of disease often have pleiotropic and overlapping phenotypes. Moreover, phenotypic descriptions in the literature in both human and mouse are often poorly characterized and difficult to compare directly.

**Methods:**

In this report, human genetic association results from the literature are summarized with regard to replication, disease phenotype, and gene specific results; and organized in the context of a systematic disease ontology. Similarly summarized mouse genetic disease models are organized within the Mammalian Phenotype ontology. Human and mouse disease and phenotype based gene sets are identified. These disease gene sets are then compared individually and in large groups through dendrogram analysis and hierarchical clustering analysis.

**Results:**

Human disease and mouse phenotype gene sets are shown to group into disease and phenotypically relevant groups at both a coarse and fine level based on gene sharing.

**Conclusion:**

This analysis provides a systematic and global perspective on the genetics of common human disease as compared to itself and in the context of mouse genetic models of disease.

## Background

Common complex diseases such as cardiovascular disease, cancer, and autoimmune disorders; metabolic conditions such as diabetes and obesity, as well as neurological and psychiatric disorders make up a majority of health morbidity and mortality in developed countries. The specific genetic contributions to disease etiology and relationships to environmental factors in common disorders are unclear; complicated by many factors such as gene-gene interactions, the balance between susceptibility and protective alleles, copy number variation, low relative risk contributed by each gene, and a myriad of complex environmental inputs.

Genetic association studies using a candidate gene approach and more recently whole genome association studies (GWAS) have produced a large and rapidly increasing amount of information on the genetics of common disease. In parallel, mouse genetic models for human disease have provided a wealth of genetic and phenotypic information. While not always perfect models for human common complex disorders, the genetic purity and experimental flexibility of mouse disease models have produced valuable insights relevant to human disease.

Gene nomenclature standardization[1], database efforts [2-4], and phenotype ontology projects[5] in both human and mouse over the past decade have provided the foundation for integration of information on genetic contributions to disease and phenotypes. This allows the opportunity for systematic comparison and higher order systems analysis of disease and phenotypic information. In this report, we summarize and integrate large scale information on human genetic association information and mouse genetically determined phenotypic information with the goal of identifying fundamental relationships in human disease and mouse models of human disease.

## Methods

### The Genetic Association Database

The Genetic Association Database [2] (GAD) http://geneticassociationdb.nih.gov is an archive of summary data of published human genetic association studies of many common disease types. GAD is primarily focused on archiving information on common complex human disease rather than rare Mendelian disorders as found in the Online Mendelian Inheritance in Man (OMIM)[6]. GAD contains curated information on candidate gene studies and more recently on genome wide association studies. It builds on the curation of the CDC HuGENet info literature database [3] in part by adding molecular and ontological annotation creating a bridge between epidemiological and molecular information. This allows the large-scale integration of disease based genetic association information with genomic and molecular information as well as with the software tools and computational approaches and that use genomic information [7-12]. This report is a summary and analysis of the genes and diseases with positive associations in the Genetic Association Database with regard to replication, comparisons between diseases, and within broad phenotypic disease classes. Although GAD contains information on gene variation, this report is at the gene level only and does not consider specific gene variation or genetic polymorphism.

The Genetic Association Database (GAD) currently contains approximately 40,000 individual gene records of genetic association studies taken from over 23,000 independent publications. Importantly, a large number (11,568) of the records in GAD have a designation of whether the gene of record was reported to be associated (Y) or was not (N) associated with the disease phenotype for that specific record. Many records, for various reasons, do not have such a designation. In addition, a portion of the database records have been annotated with standardized disease phenotype keywords from the MeSH http://www.nlm.nih.gov/mesh/ vocabulary. The GAD summations shown below are a subset of the records in GAD. They only include those records that are both; a) positively associated with a disease phenotype, and b) have a MeSH disease phenotype annotation. This represents a subset of 10,324 records having both positive associations to disease and records with MeSH annotations. Records designated as not associated (N) with a disease phenotype and those without MeSH disease annotation are not considered at this time in this report.

### Mouse phenotypic database

The mouse phenotypic information described here was obtained from the Mouse Genome Informatics (MGI) database [4]http://www.informatics.jax.org/ Phenotypes, Alleles and Disease Models section. The file used for mouse phenotypic information (see methods) is comprised of 5011 unique genes and 5142 unique phenotypic terms derived from information from specific gene mutations in multiple mouse strains. The mouse phenotypic information had been annotated to the mouse gene mutation records using Mammalian Phenotype terms and codes in the mouse phenotype database as a component of the Mouse Phenotyping Project [5,13].

### Quantitation of genes and disease phenotypes

Quantitation of how often a disease phenotype was positively associated with a gene was performed as follows. GAD records having both recorded positive associations and annotated MeSH disease keywords were extracted and stored in a database according to their relationships. Using a perl script, the number of times of co-occurrence of a MeSH disease keyword was positively associated with a specific gene was recorded as found in the GAD database. These counts were sorted in declining order for each unique gene grouped by the disease MeSH term with which they are associated.

### Mouse phenotypic information

The mouse phenotypic information described here was obtained from the Mouse Genome Informatics (MGI) http://www.informatics.jax.org/; Phenotypes, Alleles and Disease Models section; ftp://ftp.informatics.jax.org/pub/reports/index.html#pheno

Using these three files downloaded on 4-4-2008

ftp://ftp.informatics.jax.org/pub/reports/MPheno_OBO.ontology

ftp://ftp.informatics.jax.org/pub/reports/MGI_PhenotypicAllele.rpt

ftp://ftp.informatics.jax.org/pub/reports/MGI_PhenoGenoMP.rpt

The mouse phenotype files were extracted using a perl script annotating each gene with the phenotype term associated with each Mammalian Phenotype (MP) code.

### Venn Diagram overlap of individual gene lists

Individual GAD primary gene sets were analyzed using Venny[14]http://bioinfogp.cnb.csic.es/tools/venny/index.html. Pathway Venn Diagram comparisons were performed by placing individual GAD primary gene sets into WebGestalt [15]http://bioinfo.vanderbilt.edu/webgestalt/ to identify KEGG pathways, then placing the resulting pathway names into Venny.

### Dendrogram analysis of gene sets

Relationships between diseases were identified by a unique method similar to phyologenetic classification. First the distance between the diseases were calculated by pairwise comparison of the diseases by finding the common genes between the pairs and dividing it by the smallest group of the pair. This number was then subtracted from 1. This step was done because if two lists are identical (100% match) then the resultant distance should be 0. This is represented in the formula:

Where: *C*_*k*_: Genes in each disease set (where *k *= *i*, *j*); N(*C*_*k*_): Number of genes in each disease set (where *k *= *i*, *j*); d_ij _is the pairwise distance; *i, j*: index of genes in each disease set where; *i *= 1, 2, 3, ........., *n*; *j *= 1, 2, 3, ........., *m*

The disease relationships were calculated from the distance matrix using the Fitch program from the Phylip package[16]. It calculates the relationships based on the Fitch and Margoliash method of constructing the phylogenetic trees[17] using the following formula (from the Phylip manual):

where *D *is the observed distance between gene sets *i *and *j *and *d *is the expected distance, computed as the sum of the lengths of the segments of the tree from gene set *i *to gene set *j*. The quantity *n *is the number of times each distance has been replicated. In simple cases *n *is taken to be one. If *n *is chosen more than 1, the distance is then assumed to be a mean of those replicates. The power *P *is what distinguished between the Fitch and Neighbor-Joining methods. For the Fitch-Margoliash method P is 2.0 and for Neighbor-Joining method it is 0.0. As running Fitch took a long time when the gene-set size was huge (weeks for the human gene-sets and months for the mouse gene-sets), Neighbor-Joining method was used to create the replicate dendrograms (not shown) after randomizing the input order for greater confidence. The resulting coefficient matrix files were displayed using the Phylodraw graphics program[18].

### Hierarchical clustering of gene sets

Ward's minimum variance method[19] was used to find the distance between two diseases. The distance between the clusters is the ANOVA sum of squares between the two clusters added up over all the variables. At each generation, the within-cluster sum of squares is minimized over all partitions obtainable by merging two clusters from the previous generation. Ward's method joins clusters to maximize the likelihood at each level of the hierarchy under the assumptions of multivariate normal mixtures, spherical covariance matrices, and equal sampling probabilities. Distance for Ward's method is:  (taken from JMP Manual) where N_K _is the number of observations in C_K _(which is the Kth cluster, subset of {1, 2, ..., n) where n is the number of observations).  is the mean vector for cluster C_K_.

## Results

Each record in GAD represents a specific gene from a unique publication of a human population based genetic association study and is categorized into one of 24 general disease classes corresponding to broad MeSH disease or disease phenotypic groupings. Table 1 is a summary of the number of positively associated human genes in each MeSH human disease class. As represented by these disease classes the GAD database covers a broad selection of diseases falling into major disease classes including; aging studies, cancer, immune disorders, psychiatric diseases, metabolic conditions, pharmacogenomic studies, and studies of chemical dependency, among others. Similarly, each record in the phenotype files from the MGI phenotype database represents a unique mouse gene specific genetic model. Table 2 shows the general categories represented by the mouse phenotype summary files and the number of mouse genes found in each top level phenotype class. The mouse files contain a greater number of intermediate developmental and morphological phenotypes (e.g. insulin resistance, absent CD4+ T cells, abnormal spatial learning) while the human files tend to comprise a greater number of end stage clinical disease phenotypes (e.g. Type 2 Diabetes, multiple sclerosis, autism).

**Table 1:** Number of human genes associated in each Disease Class

DISEASE CLASS	# of human genes in each disease class
Neoplasms	1835
Cardiovascular Diseases	1112
Pathological Conditions, Signs and Symptoms	938
Nervous System Diseases	902
Nutritional and Metabolic Diseases	838
Mental Disorders	554
Digestive System Diseases	407
Male Urogenital Diseases	396
Musculoskeletal Diseases	366
Respiratory Tract Diseases	362
Bacterial Infections and Mycoses	256
Disorders of Environmental Origin	243
Female Urogenital Diseases and Pregnancy Complications	226
Virus Diseases	224
Skin and Connective Tissue Diseases	212
Hemic and Lymphatic Diseases	183
Eye Diseases	176
Congenital, Hereditary, and Neonatal Diseases and Abnormalities	142
Stomatognathic Diseases	130
Immune System Diseases	116
Endocrine System Diseases	98
Parasitic Diseases	57
Otorhinolaryngologic Diseases	35
Animal Diseases	4

**Table 2:** Number of Mouse genes in each General Phenotypic Class

PHENOTYPIC CLASS	# of Mouse genes in each class
unassigned top level	19186
nervous system phenotype	8149
immune system phenotype	6414
homeostasis/metabolism phenotype	5976
skeleton phenotype	5559
growth/size phenotype	5556
behavior/neurological phenotype	5417
cardiovascular system phenotype	5221
hematopoietic system phenotype	5163
reproductive system phenotype	4762
lethality-prenatal/perinatal	4409
embryogenesis phenotype	3416
skin/coat/nails phenotype	3048
vision/eye phenotype	2710
hearing/vestibular/ear phenotype	2447
muscle phenotype	2370
cellular phenotype	2335
normal phenotype	2120
renal/urinary system phenotype	2104
endocrine/exocrine gland phenotype	1871
life span-post-weaning/aging	1857
respiratory system phenotype	1832
digestive/alimentary phenotype	1780
lethality-postnatal	1777
liver/biliary system phenotype	1498
limbs/digits/tail phenotype	1282
tumorigenesis	1268
adipose tissue phenotype	1067
craniofacial phenotype	1016
pigmentation phenotype	634
touch/vibrissae phenotype	625
no phenotypic analysis	403
other phenotype	343
taste/olfaction phenotype	156

Table 3 introduces examples of human genes from fundamental biological pathways that have been consistently associated with major disease phenotypes highlighting the sometimes-broad pleiotropic effects that major regulatory molecules have on multiple disease phenotypes. Genes such as *NOS3*, nitric oxide synthase 3, regulating nitrous oxide production; *HLA-DQB1*, the MHC class II molecule DQ beta 1, involved in antigen presentation; *ACE*, angiotensin I converting enzyme, central to the renin-angiotensin system and *PPARG*, peroxisome proliferator-activated receptor gamma, regulating transcription in pathways important in lipid metabolism are examples of genes that affect multiple tissues and different organ systems through the complex course of disease progression. Importantly, all the mouse orthologs of the human genes in Table 3 have experimentally determined phenotypes that are similar or broadly overlapping with human clinical disease phenotypes (see below).

**Table 3:** Selected Major Genes and Disease Phenotypes

Gene		Gene	
**APOE**	ALZHEIMER DISEASE (70)	**VDR**	PROSTATIC NEOPLASMS (10)
	CORONARY DISEASE (8)		OSTEOPOROSIS, POSTMENOPAUSAL (8)
	CARDIOVASCULAR DISEASES (7)		BREAST NEOPLASMS (7)
	MYOCARDIAL INFARCTION (6)		DIABETES MELLITUS, TYPE 1 (6)
	DIABETES MELLITUS, TYPE 2 (6)		OSTEOPOROSIS (6)
			
**ACE**	HYPERTENSION (47)	**MTHFR**	NEURAL TUBE DEFECTS (6)
	DIABETES MELLITUS, TYPE 2 (25)		COLORECTAL NEOPLASMS (5)
	MYOCARDIAL INFARCTION (17)		DIABETES MELLITUS, TYPE 2 (5)
	CORONARY DISEASE (16)		ESOPHAGEAL NEOPLASMS (5)
	DIABETIC NEPHROPATHIES (15)		ADENOCARCINOMA (4)
			
**HLA-DQB1**	DIABETES MELLITUS, TYPE 1 (30)	**CYP17A1**	BREAST NEOPLASMS (10)
	PAPILLOMAVIRUS INFECTIONS (7)		PROSTATIC NEOPLASMS (9)
	CELIAC DISEASE (6)		PROSTATIC HYPERPLASIA (4)
	AUTOIMMUNE DISEASES (5)		OSTEOPOROSIS, POSTMENOPAUSAL (3)
	TUBERCULOSIS, PULMONARY (5)		ENDOMETRIAL NEOPLASMS (2)
			
**DRD2**	ALCOHOLISM (17)	**ADRB2**	ASTHMA (12)
	SCHIZOPHRENIA (14)		OBESITY (10)
	PERSONALITY DISORDER (2)		HYPERTENSION (8)
	DEPRESSIVE DISORDER (2)		DIABETES MELLITUS, TYPE 2 (4)
	DYSKINESIA, DRUG INDUCED (2)		BRONCHIAL HYPERREACTIVITY (4)
			
**PPARG**	DIABETES MELLITUS, TYPE 2 (18)	**NOS3**	HYPERTENSION (20)
	OBESITY (11)		MYOCARDIAL INFARCTION (18)
	DIABETES MELLITUS (6)		CORONARY ARTERY DISEASE (15)
	INSULIN RESISTANCE (4)		CORONARY DISEASE (12)
	GLUCOSE INTOLERANCE (2)		DIABETES MELLITUS, TYPE 2 (10)

### Summaries of genes and phenotypes in human and mouse

The majority of this report is built upon large non-redundant general summary lists for both human and mouse, shown below. These lists take two complimentary forms in both human and mouse. The first sets are GENE-to-Disease/Phenotype lists. These are non-redundant lists of genes showing the diseases or phenotypes that have been associated with each gene (Table 4 human, table 5 mouse, and table 6 human-mouse). The second sets of basic lists are DISEASE/PHENOTYPE-to-Gene lists. These are non redundant lists of diseases or phenotypes with the genes that have been associated with that disease or phenotype (Table 7 human and table 8 mouse).

**Table 4:** Selected Human Genes and Disease Phenotype (MeSH counts), positive associations

Gene ID	HUGO Gene Sym.	MESH TERM 1	MESH TERM 2	MESH TERM 3	MESH TERM 4
348	**APOE**	Alzheimer Disease(70)	Coronary Disease(8)	Cardiovascular Diseases(7)	Diabetes Mellitus, Type 2(6)
1636	**ACE**	Hypertension(47)	Diabetes Mellitus, Type 2(25)	Myocardial Infarction(17)	Coronary Disease(16)
3119	**HLA-DQB1**	Diabetes Mellitus, Type 1(30)	Papillomavirus Infections(7)	Celiac Disease(6)	Tuberculosis, Pulmonary(5)
1493	**CTLA4**	Diabetes Mellitus, Type 1(28)	Graves Disease(21)	Thyroiditis, Autoimmune(10)	Autoimmune Diseases(8)
183	**AGT**	Hypertension(24)	Coronary Disease(6)	Diabetic Nephropathies(5)	Myocardial Infarction(5)
1814	**DRD3**	Schizophrenia(24)	Dyskinesia, Drug-Induced(6)	Psychotic Disorders(5)	Alcoholism(2)
4846	**NOS3**	Hypertension(20)	Myocardial Infarction(18)	Coronary Artery Disease(15)	Coronary Disease(12)
3075	**CFH**	Macular Degeneration(19)	Choroidal Neovascularization(3)	Hemolytic-Uremic Syndrome(2)	Atrophy(2)
3077	**HFE**	Hemochromatosis(18)	Cardiovascular Diseases(1)	Colorectal Neoplasms(1)	Liver Cirrhosis(1)
3356	**HTR2A**	Schizophrenia(18)	Alzheimer Disease(4)	Depressive Disorder(4)	Depressive Disorder, Major(4)
1585	**CYP11B2**	Hypertension(18)	Cardiovascular Diseases(2)	Ventricular Dysfunction, Left(2)	Cardiomyopathy, Dilated(2)
5468	**PPARG**	Diabetes Mellitus, Type 2(18)	Obesity(11)	Diabetes Mellitus(6)	Insulin Resistance(4)
2784	**GNB3**	Hypertension(18)	Insulin Resistance(4)	Diabetes Mellitus, Type 2(3)	Obesity(3)
1815	**DRD4**	Attention Def. Dis. with Hyperact. (17)	Schizophrenia(8)	Substance-Related Disorders(4)	Mood Disorders(4)
1813	**DRD2**	Alcoholism(17)	Schizophrenia(14)	Personality Disorders(2)	Depressive Disorder(2)
155	**ADRB3**	Obesity(17)	Diabetes Mellitus, Type 2(9)	Insulin Resistance(6)	Endometrial Neoplasms(2)
9370	**ADIPOQ**	Diabetes Mellitus, Type 2(17)	Insulin Resistance(11)	Obesity(8)	Hypertension(4)
3123	**HLA-DRB1**	Arthritis, Rheumatoid(16)	Diabetes Mellitus, Type 1(16)	Multiple Sclerosis(8)	Lupus Erythematosus, Systemic(7)
118	**ADD1**	Hypertension(16)	Cardiovascular Diseases(3)	Cerebral Hemorrhage(1)	Diabetic Angiopathies(1)
3117	**HLA-DQA1**	Diabetes Mellitus, Type 1(15)	Graves Disease(4)	Autoimmune Diseases(4)	Celiac Disease(4)
1956	**EGFR**	Lung Neoplasms(15)	Carcinoma, Non-SC Lung(10)	Adenocarcinoma(6)	Neoplasm Recurrence, Local(3)
6690	**SPINK1**	Pancreatitis(15)	Chronic Disease(11)	Acute Disease(3)	Pancreatitis, Alcoholic(3)
6934	**TCF7L2**	Diabetes Mellitus, Type 2(15)	Insulin Resistance(4)	Diabetes Mellitus(2)	Liver Neoplasms(1)
1234	**CCR5**	HIV Infections(14)	Diabetes Mellitus, Type 2(4)	Diabetic Nephropathies(4)	Asthma(3)
5663	**PSEN1**	Alzheimer Disease(14)	Down Syndrome(2)	Dementia(1)	Cerebral Amyloid Angiopathy(1)
11132	**CAPN10**	Diabetes Mellitus, Type 2(14)	Insulin Resistance(3)	Polycystic Ovary Syndrome(2)	Obesity(2)
3553	**IL1B**	Stomach Neoplasms(14)	Helicobacter Infections(6)	Alzheimer Disease(5)	Periodontitis(3)
6532	**SLC6A4**	Depressive Disorder, Major(13)	Depressive Disorder(13)	Bipolar Disorder(10)	Alcoholism(8)
4210	**MEFV**	Familial Mediterranean Fever(13)	Amyloidosis(4)	Behcet Syndrome(3)	Colitis, Ulcerative(2)
3172	**HNF4A**	Diabetes Mellitus, Type 2(13)	Glucose Intolerance(2)	Birth Weight(1)	Fetal Macrosomia(1)
7157	**TP53**	Carcinoma, Squamous Cell(13)	Lung Neoplasms(12)	Breast Neoplasms(10)	Carcinoma, Non-SC Lung(9)
672	**BRCA1**	Breast Neoplasms(12)	Ovarian Neoplasms(5)	Carcinoma, Endometrioid(1)	DNA Damage(1)
185	**AGTR1**	Hypertension(12)	Myocardial Infarction(3)	Coronary Disease(3)	Pregnancy Comp., Cardiovascular(2)
154	**ADRB2**	Asthma(12)	Obesity(10)	Hypertension(8)	Diabetes Mellitus, Type 2(4)
3953	**LEPR**	Obesity(12)	Body Weight(4)	Insulin Resistance(4)	Glucose Intolerance(3)
2169	**FABP2**	Diabetes Mellitus, Type 2(12)	Insulin Resistance(10)	Obesity(7)	Hyperlipidemias(4)
929	**CD14**	Asthma(12)	Myocardial Infarction(5)	Arteriosclerosis(4)	Colitis, Ulcerative(4)
26191	**PTPN22**	Arthritis, Rheumatoid(11)	Diabetes Mellitus, Type 1(9)	Lupus Erythematosus, Systemic(5)	Arthritis, Psoriatic(2)
3596	**IL13**	Asthma(11)	Hypersensitivity, Immediate(4)	Pulmonary Dis., Chronic Obstr. (4)	Respiratory Hypersensitivity(2)
1080	**CFTR**	Cystic Fibrosis(10)	Pancreatitis(5)	Chronic Disease(3)	Acute Disease(2)

**Table 5:** Selected Mouse Genes-Disease Phenotypes

Mouse Gene Sym.	Human Ortholog Gene Sym.	Mouse Phenotype 1	Mouse Phenotype 2	Mouse Phenotype 3	Mouse Phenotype 4	Mouse Phenotype 5
A4galt	A4GALT	abnormal induced morb./mort.	abnormal resp./metab. to xenobiotics	life span-post-weaning/aging	homeostasis/metab. phenotype	
Abca2	ABCA2	tremors	decreased body weight	behavior/neurological phenotype	hyperactivity	increased startle reflex
Abcc2	ABCC2	abnormal blood chemistry	abnormal liver physiology	abnormal urine chemistry	abnormal kidney physiology	Abn. resp./metabolism to xenobiotics
Abi2	ABI2	abn. corpus callosum morph.	abnormal cerebral cortex morph.	abnormal hippocampus morph.	abnormal dentate gyrus morph.	microphthalmia
Acaca	ACACA	abnormal liver physiology	abnormal lipid level	incr. circulating free fatty acid level	hyperglycemia	embryonic growth arrest
Acads	ACADS	hypoglycemia	behavior/neurological phenotype	abnormal drinking behavior	abnormal food preference	abnormal urine chemistry
Accn1	ACCN1	retinal degeneration	vision/eye phenotype	abnormal eye electrophysiology		
Adad1	ADAD1	impaired fertilization	male infertility	asthenozoospermia	oligozoospermia	reproductive system phenotype
Adam23	ADAM23	tremors	behavior/neurological phenotype	ataxia	postnatal lethality	lethality-postnatal
Adarb1	ADARB1	behavior/neurological phenot.	seizures	postnatal lethality	behavior/neurological phenotype	normal phenotype
Adipoq	ADIPOQ	vasculature congestion	increased body weight	decreased body weight	abnormal CNS syn. transmission	abnormal coat appearance
Adora1	ADORA1	behavior/neurological phenot.	increased anxiety-related response	abnormal body temperature regulation	abnormal angiogenesis	abnormal nervous system electrophys.
Ager	AGER	increased bone density	abnormal cancellous bone morph.	abnormal blood chemistry	reproductive system phenotype	abnormal cell proliferation
Akap1	AKAP1	reduced female fertility	decreased litter size	abnormal female meiosis	increased cholesterol level	
Apoc1	APOC1	abnormal circ. cholesterol level	abnormal lipid level	increased circulating triglyceride level	abnormal immune sys. Morph.	abnormal bile composition
B2m	B2M	decreased hematocrit	abnormal interleukin-10 physiology	rectal prolapse	abnormal dorsal root gang. morph.	enlarged spleen
Bax	BAX	enlarged spleen	increased thymocyte number	abnormal motor neuron morph.	short snout	abnormal sympathetic neuron morph.
Bcl2	BCL2	small ears	absent melanin granules in hair follicle	abnormal snout morph.	herniated abdominal wall	abnormal small intestine morph.
Bmp1	BMP1	abnormal heart morph.	abnormal aorta morph.	abnormal ventricular septum morph.	abnormal awl hair	prenatal lethality
Brca1	BRCA1	abnormal cell death	increased cell proliferation	decreased cell proliferation	decreased anxiety-related resp.	kinked tail
Capn10	CAPN10	abnormal pancreas physiology	endocrine/exocrine gland phenotype	digestive/alimentary phenotype	decreased inflammatory response	
Casp1	CASP1	abnormal apoptosis	abnormal induced morbidity/mortality	abnormal inflammatory response	decr. suscep. to endotoxin shock	tumorigenesis
Ccr4	CCR4	immune system phenotype	decreased tumor necrosis factor secr.	decreased interleukin-1 beta secretion	abnormal induced morbid./mort.	
Dusp1	DUSP1	thick alveolar septum	abnormal circ. alanine transaminase	hypotension	increased thymocyte number	lung inflammation
E2f1	E2F1	abnormal cell death	decreased salivation	enlarged thymus	pale liver	exencephaly
Epo	EPO	abnormal erythropoiesis	abnormal pericardium morph.	small liver	postnatal growth retardation	abnormal hepatocyte morph.
Ercc4	ERCC4	abnormal cell content/morph.	abnormal liver morph.	decreased body weight	absent blood islands	liver/biliary system phenotype
F5	F5	behavior/neurological phenot.	abnormal somite development	abnormal yolk sac morph.	increased suscep. to bact. Infect.	hemorrhage
Fcgr1	FCGR1A	impaired macrophage phagocyt.	abnormal inflammatory response	decreased inflammatory response	abnormal yolk sac morph.	abnormal cell-mediated immunity
Foxo1	FOXO1	absent organized vascular net.	abnormal looping morphogenesis	abnormal vasculature	exencephaly	absent vitelline blood vessels
Gadd45a	GADD45A	decreased leukocyte cell num.	increased cell proliferation	increased thymocyte number	postnatal lethality	skin irradiation sensitivity
Gap43	GAP43	decreased body weight	abnormal optic nerve innervation	absent optic tract	abnormal erythropoiesis	nervous system phenotype
Gata1	GATA1	decreased hematocrit	abnormal thrombopoiesis	extramedullary hematopoiesis	overexpanded resp. alveoli	liver hypoplasia
Grin1	GRIN1	abn. trigeminal nerve morph.	atelectasis	lung hemorrhage	abnormal tympanic ring morph.	decreased body weight
Hoxa1	HOXA1	small ears	abnormal inner ear morph.	abnormal malleus morph.	increased susceptibility to injury	abnormal cochlea morph.
Hspa1a	HSPA1A	decreased body weight	increased cell. Sens. to gamma-irrad.	chromosome breakage	increased body weight	homeostasis/metabolism phenotype
Icam1	ICAM1	increased leukocyte cell number	increased neutrophil cell number	increased monocyte cell number	abnormal spatial learning	abnormal retina morph.
Igbp1	IGBP1	decreased thymocyte number	behavior/neurological phenotype	abnormal cued conditioning behavior	intestinal ulcer	abnormal thymus lobule morph.

**Table 6:** Selected Human-Mouse Phenotype Overlap

Mouse Gene Sym	Human Gene Sym	Human Gene ID #	Human Disease MeshTerm	Mouse Phenotype Term
**Npc1l1**	**NPC1L1**	29881	Hypercholesterolemia(1)	abnormal circulating LDL cholesterol level; decreased circulating HDL cholesterol level; abnormal triglyceride level; abnormal lipid homeostasis; ...
				
**Nkx2-5**	**NKX2-5**	1482	Heart Defects, Congenital(1), Heart Block(1)	abnormal heart development; abnormal looping morphogenesis; abnormal heart tube morphology; abnormal heart shape; thin ventricular wall; ...
				
**Oprm1**	**OPRM1**	4988	Alcoholism(9), Substance-Related Disorders(5), Heroin Dependence(2), Pain, Postoperative(2), Epilepsy, Generalized(1), Substance Withdrawal Syndrome(1), Cocaine-Related Disorders(1), Diabetes Mellitus, Type 2(1), Kidney Failure, Chronic(1), Pain(1), Ischemia(1), Opioid-Related Disorders(1), Postoperative Nausea and Vomiting(1)	abnormal response to addictive substance; preference for addictive substance; abnormal touch/nociception; abnormal pain threshold; decreased chemically-elicited antinociception; sensitivity to addictive substance; excitatory postsyn. potential; resistance to addictive substance; altered response to anesthetics; ...
				
**Homer1**	**HOMER1**	9456	Cocaine-Related Disorders(1)	cocaine preference; abnormal conditioning behavior; abnormal response to addictive substance; nervous system phenotype; abnormal nervous system physiology; behavior/neurological phenotype, ...
				
**Insl3**	**INSL3**	3640	Cryptorchidism(3), Abnormalities, Multiple(1), Hypospadias(1), Gonadal Dysgenesis(1), Infertility, Male(1), Testicular Diseases(1)	abnormal male reproductive anatomy; small testis; abnormal spermatogenesis; behavior/neurological phenotype; male infertility; female infertility; abnormal estrous cycle; abnormal gametogenesis; decreased germ cell number; cryptorchism; ...
				
**Stat6**	**STAT6**	6778	Asthma(3), Hypersensitivity(3), Dermatitis, Atopic(2), Anaphylaxis(2), Nut Hypersensitivity(1), Nephrotic Syndrome(1), Infertility(1), Hypersensitivity, Immediate(1), Graves Disease(1), Endometriosis(1), ...	abnormal humoral immune response; decreased IgM level; decreased IgA level; decreased susceptibility to viral infection; decreased IgE level; increased IgG level; increased IgA level; abnormal interleukin physiology; abnormal interferon physiology; abnormal CD8-positive T cell morphology; ...
				
**En2**	**EN2**	2020	Autistic Disorder(1), Asperger Syndrome(1)	abnormal social investigation; abnormal spatial learning; abnormal social/conspecific interaction; abnormal cerebellum morphology; abnormal cerebellar foliation; abnormal vermis morphology; abnormal cerebellar granule layer; abnormal colliculi morphology; hyperactivity; impaired coordination; abnormal grooming behavior; ...
				
**Hsd11b1**	**HSD11B1**	3290	Diabetes Mellitus, Type 2(2), Obesity(2), Hypertension(2), Insulin Resistance(2), Polycystic Ovary Syndrome(1), Hyperandrogenism(1)	abnormal abdominal fat pads; abnormal circulating cholesterol level; decreased circulating LDL cholesterol level; enlarged adrenal glands; increased circulating HDL cholesterol level; abnormal glucose homeostasis; decreased circulating triglyceride level; abnormal corticosterone level; improved glucose tolerance; ...
				
**Msh3**	**MSH3**	4437	Lung Neoplasms(1), Head and Neck Neoplasms(1), Colonic Neoplasms(1), Carcinoma, Squamous Cell(1), Carcinoma, Small Cell(1)	tumorigenesis; increased tumor incidence; premature death; life span-post-weaning/aging
				
**Crb1**	**CRB1**	23418	Optic Atrophies, Hereditary(1), Blindness(1)	abnormal retinal photoreceptor morphology; abnormal retina morphology; retinal degeneration; decreased retinal photoreceptor cell number; photosensitivity; abnormal ocular fundus morphology; nervous system phenotype; abnormal retinal photoreceptor layer; abnormal photoreceptor inner segment morph; ...
				
**Chrna7**	**CHRNA7**	1139	Schizophrenia(3), Auditory Perceptual Disorders(1), Memory Disorders(1)	pharmacologically induced seizures; decreased anxiety-related response; abnormal spatial learning; abnormal hippocampus function; abnormal tumor necrosis factor physiology; homeostasis/metabolism phenotype
				
**Inha**	**INHA**	3623	Ovarian Failure, Premature(2), Amenorrhea(1)	kyphoscoliosis; abnormal liver morphology; abnormal ovarian follicle morphology; enlarged testes; abnormal spermatogenesis; increased circulating follicle stimulating hormone; male infertility; female infertility; tumorigenesis; ovary hemorrhage; cachexia; diffuse hepatic necrosis; pancytopenia; liver/biliary system phenotype; ...
				
**Slc6a3**	**SLC6A3**	6531	Attention Deficit Disorder w/Hyp.(7), Tobacco Use Disorder(3), Schizophrenia(2), Alcohol Withdrawal Delirium(2), Eating Disorders(1), Substance Withdrawal Syndrome(1), Stress Disorders, Post-Traumatic(1), Child Behavior Disorders(1), Bulimia(1), Alcoholism(1),	abnormal maternal nurturing; hyperactivity; hypoactivity; impaired coordination; increased exploration in new environment; decreased exploration in new environment; abnormal spatial learning; abnormal pituitary secretion; abnormal lactation; increased dopamine level; cocaine preference; ...
				
**Cyp11b2**	**CYP11B2**	1585	Hypertension(18), Cardiovascular Diseases(2), Ventricular Dysfunction, Left(2), Cardiomyopathy, Dilated(2), Arteriosclerosis(1), Acromegaly(1), Fibrosis(1), Arthritis, Rheumatoid(1), Polycystic Ovary Syndrome(1), Metabolic Syndrome X(1),	decreased body size; hypotension; increased circulating corticosterone level; decreased circulating aldosterone level; decreased circulating chloride level; increased circulating renin level; abnormal enzyme/coenzyme level; lethality-postnatal; homeostasis/metabolism phenotype; growth/size phenotype; ...
				
**Ptpn22**	**PTPN22**	26191	Arthritis, Rheumatoid(11), Diabetes Mellitus, Type 1(9), Lupus Erythematosus, Systemic(5), Arthritis, Psoriatic(2), Autoimmune Diseases(2), Arthritis, Juvenile Rheumatoid(2), Nephritis(1), Multiple Sclerosis(1), Asthma(1), Cholangitis, Sclerosing(1),	enlarged spleen; enlarged lymph nodes; abnormal Peyer's patch germinal center morph; abnormal T cell physiology; increased IgE level; increased B cell number; immune system phenotype; hematopoietic system phenotype; increased follicular B cell number; increased spleen germinal center number; increased IgG1 level; increased IgG2a level; ...

**Table 7:** Selected Human Disease Phenotypes (MeSH) and Gene counts, positive associations

Disease Mesh Term	Gene Rank 1	Gene Rank 2	Gene Rank 3	Gene Rank 4	Gene Rank 5	Gene Rank 6	Gene Rank 7	Gene Rank 8
**DISEASE CLASS - CARDIOVASCULAR**								
Hypertension	ACE(47)	AGT(24)	NOS3(20)	CYP11B2(18)	GNB3(18)	ADD1(16)	AGTR1(12)	ADRB2(8)
Myocardial Infarction	NOS3(18)	ACE(17)	SERPINE1(11)	ITGA2(7)	LPL(6)	APOE(6)	GP1BA(5)	F7(5)
Coronary Disease	ACE(16)	NOS3(12)	PON1(11)	APOB(11)	APOE(8)	LPL(7)	AGT(6)	SERPINE1(6)
Coronary Artery Disease	NOS3(15)	PON1(9)	ACE(7)	APOA5(6)	APOE(5)	AGT(4)	ABCA1(4)	APOA1(4)
Hypertrophy, Left Ventricular	ACE(15)	GNB3(3)	AGTR2(2)	EDN1(2)	TNNT2(2)	NOS3(2)	ENPP1(1)	ACE2(1)
Venous Thrombosis	F5(8)	F2(5)	SERPINE1(4)	MTHFR(3)	ABO(2)	F8(2)	JAK2(2)	PROCR(2)
Cardiovascular Diseases	APOE(7)	CETP(6)	ACE(5)	NOS3(5)	PON1(4)	APOA5(4)	APOC3(4)	SERPINE1(3)
Myocardial Ischemia	ACE(6)	LPL(5)	NOS3(2)	ITGB3(2)	APOB(2)	AGT(1)	AGTR1(1)	SELPLG(1)
Arteriosclerosis	ACE(5)	CD14(4)	PON1(3)	FGB(3)	MTHFR(3)	NOS3(3)	APOE(3)	TLR4(2)
Cardiomyopathies	TTR(5)	HFE(1)	APOA1(1)	HLADQB1(1)	SOD2(1)	CCR2(1)	SELE(1)	MMP9(1)
Heart Failure	ADRA2C(5)	ADRB1(5)	ACE(3)	NOS3(3)	ADRB2(3)	AMPD1(2)	SCNN1B(1)	EDN1(1)
								
**DISEASE CLASS - DIGESTIVE SYS. DISEASES**								
Pancreatitis	SPINK1(15)	CFTR(5)	PRSS1(3)	HLA-DRB1(2)	HLA-A(1)	TLR4(1)	UGT1A7(1)	KRT8(1)
Cystic Fibrosis	CFTR(10)	NOS1(2)	SERPINA1(2)	SPINK1(1)	CAPN10(1)	SFTPA2(1)	GCLC(1)	FCGR2A(1)
Celiac Disease	HLADQB1(6)	CTLA4(6)	HLADQA1(4)	TNF(2)	PTPN22(1)	IFNG(1)	TIPARP(1)	IL21(1)
Crohn Disease	IL23R(6)	NOD2(5)	TNF(5)	ABCB1(4)	CD14(4)	IBD5(3)	DLG5(3)	MIF(3)
Liver Cirrhosis, Alcoholic	ALDH2(6)	ACE(1)	TNF(1)	SOD2(1)	ADH1C(1)	ADH1B(1)	DRD2(1)	CYP2E1(1)
Colitis, Ulcerative	ABCB1(5)	IL23R(5)	TNF(4)	CD14(4)	TLR4(3)	ICAM1(3)	IL1RN(3)	CTLA4(3)
Gastritis, Atrophic	MPO(3)	TLR4(1)	IL13(1)	PTPN11(1)	TNF(1)	ABO(1)	CMA1(1)	IL1B(1)
Inflammatory Bowel Diseases	TNF(3)	ABCB1(3)	IL23R(3)	ITPA(2)	NOD2(2)	DLG5(2)	HP(2)	PON1(1)
Cholangitis, Sclerosing	HLADRB1(2)	HP(2)	PTPN22(1)	MMP1(1)	HLADQA1(1)	TNF(1)	HLADQB1(1)	MMP3(1)
								
**DISEASE CLASS - DIS. OF ENVIRONMENTAL ORIGIN**								
Alcoholism	DRD2(17)	OPRM1(9)	SLC6A4(8)	ALDH2(7)	MAOA(6)	GABRA2(4)	NPY(4)	ADH1B(3)
DNA Damage	XRCC1(7)	TP53(3)	CYP1A1(3)	GSTM1(3)	OGG1(3)	LIG4(2)	APEX1(2)	BRCA2(2)
Substance-Related Disorders	SLC6A4(5)	OPRM1(5)	DRD4(4)	DRD5(2)	BDNF(2)	ADH4(2)	CNR1(2)	DRD2(2)
Fractures, Bone	ESR1(4)	ESR2(2)	COL1A1(2)	CYP19A1(1)	IGF1(1)	TNFRSF11B(1)	P2RX7(1)	TGFB1(1)
Tobacco Use Disorder	CYP2A6(3)	SLC6A3(3)	CHRNA4(2)	TH(2)	BDNF(1)	PPP1R1B(1)	SLC18A2(1)	PTEN(1)
Cocaine-Related Disorders	PDYN(2)	HOMER1(1)	TTC12(1)	ANKK1(1)	DBH(1)	GSTP1(1)	OPRM1(1)	
Heroin Dependence	OPRM1(2)	BDNF(1)	OPRD1(1)	SLC6A4(1)	COMT(1)	MAOA(1)		
Spinal Fractures	COL1A1(2)	CYP19A1(1)	TNFRSF11B(1)	GC(1)	PLXNA2(1)	AR(1)	NOS3(1)	
								
**DISEASE CLASS - IMMUNE SYSTEM**								
Autoimmune Diseases	CTLA4(8)	HLADQB1(5)	HLADRB1(4)	HLADQA1(4)	PTPN22(2)	HLA-A(2)	CYP2D6(2)	CIITA(2)
Hypersensitivity, Immediate	IL4R(8)	IL13(4)	CD14(4)	IL4(2)	SERPINE1(2)	CCL5(2)	NOS2A(2)	CTLA4(2)
Graft vs Host Disease	IFNG(3)	TNF(2)	TLR4(1)	NOD2(1)	HLA-DPB1(1)	HLA-A(1)	IL10(1)	IL1R1(1)
Hypersensitivity	IL4(3)	STAT6(3)	IL4R(2)	IFNG(1)	IFNGR1(1)	TLR2(1)	FADS1(1)	IL13(1)
Antiphospholipid Syndrome	F2(2)	SELPLG(1)	SERPINE1(1)	FCGR2A(1)	HLADMA(1)			
Food Hypersensitivity	IL4(1)	IL4R(1)	STAT6(1)	IL13(1)	HLADQB1(1)	CD14(1)		
								
**DISEASE CLASS - MENTAL DISORDERS**								
Schizophrenia	DRD3(24)	HTR2A(18)	DRD2(14)	COMT(10)	HTR2C(8)	BDNF(8)	DRD4(8)	NOTCH4(8)
Attention Deficit Disorder with Hyperactivity	DRD4(17)	SLC6A3(7)	SLC6A4(6)	ADRA2A(4)	MAOA(3)	SNAP25(3)	SLC6A2(2)	DRD5(2)
Depressive Disorder	SLC6A4(13)	HTR2A(4)	TPH1(3)	CYP2D6(2)	CYP2C19(2)	MAOA(2)	BDNF(2)	DRD2(2)
Depressive Disorder, Major	SLC6A4(13)	TPH1(5)	HTR2A(4)	TPH2(3)	BDNF(2)	DRD2(2)	GNB3(2)	DTNBP1(1)
Bipolar Disorder	SLC6A4(10)	BDNF(6)	MAOA(5)	COMT(5)	XBP1(3)	GABRA5(3)	HTR2A(3)	TPH2(3)
Anxiety Disorders	SLC6A4(7)	MAOA(3)	PLXNA2(1)	BDNF(1)	DBI(1)	MED12(1)	GABRB3(1)	DRD2(1)
Mood Disorders	SLC6A4(5)	DRD4(4)	CLOCK(2)	MAOA(2)	BDNF(2)	ACE(1)	CRH(1)	DRD3(1)
Psychotic Disorders	DRD3(5)	SLC6A4(3)	DRD4(3)	HTR2A(3)	DTNBP1(2)	DISC1(2)	DRD2(2)	MAOA(1)
Obsessive-Compulsive Disorder	SLC6A4(4)	HTR2A(3)	COMT(3)	SLC1A1(2)	DRD4(2)	HTR1B(1)	BDNF(1)	NRCAM(1)
Panic Disorder	CCK(4)	HTR1A(2)	MAOA(2)	HTR2A(2)	DBI(1)	CCKAR(1)	ADORA2A(1)	PGR(1)
Cognition Disorders	APOE(3)	BDNF(3)	DRD4(2)	COMT(2)	HMGCR(1)	DTNBP1(1)	SLC6A4(1)	NQO1(1)
								
**DISEASE CLASS - NERVOUS SYSTEM DISEASES**								
Alzheimer Disease	APOE(70)	PSEN1(14)	A2M(10)	CYP46A1(8)	ACE(7)	BCHE(7)	IL1A(7)	BDNF(6)
Parkinson Disease	PARK2(9)	LRRK2(9)	CYP2D6(7)	MAOB(7)	BDNF(5)	SNCA(5)	PON1(4)	PINK1(4)
Multiple Sclerosis	HLADRB1(8)	APOE(5)	CTLA4(4)	PTPRC(4)	MBP(3)	HLA-DQB1(3)	IFNG(2)	CRYAB(2)
Amyotrophic Lateral Sclerosis	SOD1(6)	PON1(2)	PON2(2)	VEGFA(2)	SMN1(1)	MAPT(1)	MT-ND5(1)	PON3(1)
Brain Ischemia	FGB(5)	PDE4D(3)	NOS3(3)	ACE(2)	PON1(2)	MTHFR(2)	ITGB3(2)	TLR4(1)
Cerebrovascular Accident	NOS3(5)	APOE(5)	FGB(5)	PON1(4)	SERPINE1(3)	ALOX5AP(3)	ACE(2)	KL(2)
Carotid Artery Diseases	NOS3(4)	PON1(3)	MTHFR(3)	CCL2(2)	IL6(2)	APOE(2)	CD14(2)	ACE(1)
Dementia	APOE(4)	MAPT(3)	MT-ND1(1)	PRNP(1)	PSEN1(1)	TNF(1)	CDC2(1)	IGF1R(1)
								
**DISEASE CLASS - NUTR. AND METABOLIC DISEASES**								
Diabetes Mellitus, Type 2	ACE(25)	PPARG(18)	ADIPOQ(17)	TCF7L2(15)	CAPN10(14)	HNF4A(13)	FABP2(12)	NOS3(10)
Obesity	ADRB3(17)	LEPR(12)	MC4R(11)	PPARG(11)	UCP2(11)	ADRB2(10)	UCP1(8)	ADIPOQ(8)
Insulin Resistance	ADIPOQ(11)	FABP2(10)	INSR(7)	IRS1(7)	ENPP1(7)	ADRB3(6)	NOS3(6)	ACE(5)
Diabetes Mellitus	PPARG(6)	ACE(3)	INS(3)	NOS3(3)	PON1(2)	UBL5(2)	IRS1(2)	TCF7L2(2)
Hyperlipidemias	APOA5(5)	FABP2(4)	LPL(3)	APOE(3)	ACE(2)	APOA1(2)	PPARA(2)	PPARG(2)
Hypertriglyceridemia	APOC3(5)	APOA5(4)	LPL(3)	APOE(3)	ADRB2(2)	APOA4(2)	GP1BA(1)	LTA(1)
Glucose Intolerance	LEPR(3)	ADIPOQ(3)	IGF1(2)	KCNJ11(2)	PTPN1(2)	PPARG(2)	HNF4A(2)	NEUROG3(1)
Hypercholesterolemia	APOA1(3)	APOB(3)	F12(3)	ACE(2)	LDLR(2)	LPL(2)	PCSK9(2)	ABCG8(2)
Metabolic Syndrome	APOC3(3)	UBL5(2)	NOS3(2)	ACE(1)	PPARD(1)	NPY5R(1)	ACE2(1)	RGS2(1)
								
**DISEASE CLASS - EYE DISEASES**								
Macular Degeneration	CFH(19)	APOE(4)	PON1(2)	C2(1)	CFB(1)	ABCA1(1)	HTRA1(1)	MELAS(1)
Diabetic Retinopathy	VEGFA(7)	AKR1B1(4)	PON1(3)	RAGE(3)	AGER(3)	ACE(2)	ITGA2(2)	ICAM1(2)
Glaucoma	CYP1B1(3)	OPTN(2)	OPA1(2)	OPTC(1)	EDNRA(1)	MYOC(1)		
Ocular Hypertension	OPTN(2)	CYP1B1(1)	OLFM2(1)	OPA1(1)				
Cataract	GALT(1)	AIPL1(1)	IFNGR1(1)	GCNT2(1)				
Retinal Degeneration	NDP(1)	GUCA1A(1)	AIPL1(1)	COL2A1(1)	RHO(1)	GUCA1B(1)	ABCA4(1)	
Myopia	HLADPB1(1)	LUM(1)	COL2A1(1)	NYX(1)	MYOC(1)			

**Table 8:** Selected Mouse Disease Related Phenotypes

PhenoCode	PhenoType							
	**DISEASE CLASS CARDIOVASCULAR**	**Gene 1**	**Gene 2**	**Gene 3**	**Gene 4**	**Gene 5**	**Gene 6**	**Gene 10**
								
MP:0005048	thrombosis	Abca5	Actc1	Adamts13	Ahr	Alox12	Anxa2	F2rl2
MP:0005341	decreased sus. to atherosclerosis	APOA1	Apoe	Artles	Ath17	Ath29	Ath37	Icam1
MP:0000231	hypertension	Abcc9	Ace2	Add2	Agt	Alb1-Ren	Bpq5	Chga
MP:0004181	abnormal carotid artery morphology	Aldh1a2	Chrd	Crk	Ednra	Fgf8	Foxm1	Shc1
MP:0004111	abnormal coronary artery morph.	Adm	Ahr	Fgf8	Gja1	Hspg2	Itga4	Vegfa
MP:0005338	atherosclerotic lesions	Aorls1	Aorls2	Apoe	Ath29	Ath6	Ath8	Fabp4-Aebp1
MP:0000343	altered resp. to myocardial infarction	Agtr2	Aifm1	Ak1	Bnip3	CMV-Abcc9	Ccr1	Ckm-Prkaa2
MP:0006058	decreased cerebral infarction size	ACTB-Ngb	EGFP	Adora2a	Cx3cl1	F11	F12	Plat
MP:0003037	increased infarction size	Aifm1	Fgf2	Hmox1	Kit	Mapk1	Myh6-tTA	Thbd
MP:0004875	Inc. mean arterial blood pressure	Ddah1	Edn1	Ednrb	Kcnn3	Ptger1	Tagln-tTA	
MP:0005339	Inc. susceptibility to atherosclerosis	Apoa1	Apoe	Artles	Ascla1	Ascla2	Ascla3	Ath18
								
	**DISEASE CLASS - DIGESTIVE SYSTEM DISEASES**	**Gene 1**	**Gene 2**	**Gene 3**	**Gene 4**	**Gene 5**	**Gene 6**	**Gene 10**
								
MP:0003119	abnormal digestive system dev.	Cdkn1c	Cyp26a1	Foxp4	Mapk7	Mcm4	Nckap1	Tbx6
MP:0000462	abnormal digestive system morph.	Apc	Bmp5	Cdcs1	Cdkn1c	Cftr	Ctnnbip1	Gast
MP:0001663	abnormal digestive system phys.	Apoe	Cd44	Cftr	Clec7a	Col2a1	Fut2	Gpx1
MP:0000474	abnormal foregut morphology	Apc	Foxa2	Gata4	Gdf1	Hgs	Ldb1	Otx2
MP:0000488	abnormal intestinal epithelium morph	Atr	B4galt1	B9d2	Bdkrb2	Cbfa2t2	Col1a1	Elf3
MP:0003449	abnormal intestinal goblet cells	Areg	Cbfa2t2	Cftr	Clca3	Ctnnb1	E2f4	Il13
MP:0006001	abnormal intestinal transit time	Drd2	Gfra2	Gucy1b3	Hmox2	Mrvi1	Smtn	
MP:0000470	abnormal stomach morphology	Ahr	Aire	Barx1	Celsr3	Cfc1	Col1a1	Gdf11
								
	**DISEASE CLASS - DIS. OF ENVIRONMENTAL ORIGIN**	**Gene 1**	**Gene 2**	**Gene 3**	**Gene 4**	**Gene 5**	**Gene 6**	**Gene 10**
								
MP:0001425	abnormal alcohol consumption	Aaq1	Alcp1	Alcp19	Alcp2	Ap7q	Ap8q	Ppp1r1b
MP:0005443	abnormal ethanol metabolism	Adh1	Adh7	Afteq1	Afteq2	Alcw3	Htas2	
MP:0002552	abnormal response to addictive sub.	Adora2a	Adra1d	Alcw1	Alcw2	Alcw3	Alcw4	Chrna4
MP:0001987	alcohol preference	Alcp1	Alcp25	Alcp3	Alcp4	Alprf	Ap1q	Ap5q
MP:0001988	cocaine preference	Grm2	Homer1	Homer2	Per2	Slc6a3	Slc6a4	
MP:0003546	decreased alcohol consumption	Camk2a	Gnas	Gria3	Prkce	tmgc55		
MP:0004048	resistance to addictive substance	Adora2a	Adra1b	Apba1	Aqp4	Btbd14b	Chrna4	Slc6a3
								
	**DISEASE CLASS - IMMUNE SYSTEM DISEASES**	**Gene 1**	**Gene 2**	**Gene 3**	**Gene 4**	**Gene 5**	**Gene 6**	**Gene 10**
								
MP:0001844	autoimmune response	Tcra	Tcrb	ACTB	Aire	Cd1d1	Fas	Ikzf3
MP:0005016	decreased lymphocyte cell number	Atm	Bcl2	Bcl6b	Birc2	C3ar1	Ccr9	Ctsd
MP:0008088	abnormal T-helper 1 cell diff.	Cbfb	Ifngr2	Il2	Il4	Irf4	Mapk8	Sit1
MP:0002499	chronic inflammation	Ccr7	Gstz1	Hmox1	Il10	Il1rn	Jak3	Plcg2
MP:0004804	dec. sus. to autoimmune diabetes	HLA-DQA1	HLA-DQB1	Art2a	B2m	Cd4	Cd4DsRed	Cdk4
MP:0002411	decreased sus. to bacterial infection	Anth	Anth2	B2m	C4b	Casp1	Cd97	Dcn
MP:0005597	dec. sus. to type I hypers-reaction	Alox5	Alox5ap	Cysltr1	Cysltr2	Fcer1a	Fcer1g	Orai1
MP:0003725	increased autoantibody level	Tcra	Tcrb	Acla1	Acla2	Aire	Cd276	Cia38
MP:0005014	increased B cell number	BCL2	Bak1	Bax	Bcl11b	Bcl2l11	Bst1	Cdkn2c
MP:0005013	increased lymphocyte cell number	Axl	B4galt1	Bak1	Casp8	Cd19	Ewsr1	Galnt1
MP:0004803	Inc. sus. to autoimmune diabetes	Ins1-Cat	Tyr	B2m	Cd274	Cd28	Cd38	Cdk2
MP:0005350	Inc. sus. to autoimmune disorder	Tcra	Tcrb	Ads1	Ads2	Ads3	Ads4	Bak1
MP:0002412	increased sus. to bacterial infection	Adamts13	Adcyap1r1	Adh5	Atf2	Bbaa21	Bcl10	C3
								
	**DISEASE CLASS - MENTAL DISORDERS DISEASE CLASS - MENTAL DISORDERS**	**Gene 1**	**Gene 2**	**Gene 3**	**Gene 4**	**Gene 5**	**Gene 6**	
								
MP:0001412	excessive scratching	Atp2b4	Bdnf	Ctsl	EIF1AX	Lck-Il31ra	Mapt	**Gene 10**
MP:0001362	abnormal anxiety-related response	App	Araf	Axtofd1	Axtofd3	Axtofd4	Axtofd5	
MP:0001458	abnormal object recognition memory	Gabbr1	Gal	Grin1	Prnp	Prnp-App	Psen1	Crhr1
MP:0001360	abnormal social investigation	Avpr1a	Avpr1b	Cadps2	En2	Gnao1	Grin1	
MP:0002557	abnormal social/conspecific int.	Ar	Cadps2	Disc1	En2	Grin1	Grin3b	Maoa
MP:0002065	abnormal fear/anxiety-related beh.	APPV717I	App	Atp1a2	Crebbp	Egr1	Gnai1	Oxt
MP:0001364	decreased anxiety-related response	APP	Adcy8	Adcyap1	Adcyap1r1	Avpr1a	B3galt2	Nos3
MP:0002573	behavioral despair	Adra2c	B3gnt2	Cacna1c	Crhr2	Desp1	Desp2	Camk2a
MP:0001462	abn. avoidance learning behavior	Aal	Aap	Dcx	Idua	Ntrk2		Nr3c1
								
	**DISEASE CLASS - NUTR. AND METABOLIC DISEASES**	**Gene 1**	**Gene 2**	**Gene 3**	**Gene 4**	**Gene 5**	**Gene 6**	
								
MP:0005560	decreased circulating glucose level	Ins1-Cat	Tyr	Acadm	Adipoq	Apcs-Lep	Apoe	**Gene 10**
MP:0004185	abnormal adipocyte glucose uptake	Akt2	Bglap1	Cebpa	Pik3r1	Prkci	Ptprv	Cd36
MP:0000188	abnormal circulating glucose level	Adipor1	Cidea	Ciita	Ckm	Crh	Dbm3	
MP:0001560	abnormal circulating insulin level	Cacna1c	Cebpa	Foxa1	Gal	Gck	IGFBP2	Irs2
MP:0003383	abnormal gluconeogenesis	Adipoq	Adipor1	Cebpa	Cebpb	Lpin1	Mc2r	Mgat4a
MP:0005291	abnormal glucose tolerance	Adipoq	Fstl3	Irs4	Lep	Pcsk1	Pnpla2	Smarcb1
MP:0003564	abnormal insulin secretion	Eif2ak3	Gast	Gck	Gjd2	Ins2	Lep	
MP:0002727	decreased circulating insulin level	Adcyap1r1	Adipor2	Ahsg	Akt2	Apcs-Lep	Apoa2	
MP:0002711	decreased glucagon secretion	Cacna1e	Dbh	Kcnj11	Nkx2-2	Pcsk2		Bglap1
MP:0003059	decreased insulin secretion	Abcc8	Anxa7	Bglap1	Cacna1e	Cartpt	Chrm3	
MP:0001548	hyperlipidemia	APOC1	Acox1	Apc	Apoe	Cdkn1b	Cpt1c	Eif2s1
MP:0005293	impaired glucose tolerance	APPswe	PSEN1dE9	Abcc8	Acadvl	Adcyap1r1	Adipoq	Lepr
MP:0005292	improved glucose tolerance	Adipor2	Ahsg	Bcat2	Cbl	Crebbp	Cxcl14	Akt2
MP:0004892	increased adiponectin level	Actb	Adipor2	Cideb	Crebbp	Pde3b	Pten	Gcgr
MP:0002575	Inc. circulating ketone body level	Acacb	Adcyap1	Gck	AZIP	Ins2	Ins2-Nos2	Scd1
MP:0003645	Inc. pancreatic beta cell number	ACTB	Akt2	Arx	Hnf4a	Cdkn1b	Foxo1	Ins2-rtTA
MP:0001759	increased urine glucose level	Aqp1	Aqp7	Cdk4	Cdk4	Cryaa-TAg	Dnajc3a	Ins1
MP:0005331	insulin resistance	APOB	Adipoq	Adipor1	Clcn5	Adra1b	Akt2	Bglap1
								
	**DISEASE CLASS - EYE DISEASES**	**Gene 1**	**Gene 2**	**Gene 3**	**Gene 4**	**Gene 5**	**Gene 6**	**Gene 10**
								
MP:0001299	abnormal eye distance/position	Dst	Edg2	Hectd1	Hesx1	Itgb1	Nrtn	
MP:0000776	abnormal inferior colliculus	Atg5	En1	Ext1	Fgf17	Fgf8	Fgfr1	
MP:0003236	abnormal lens capsule morphology	Abi2	Cdkn2a	Cryaa	Cryga	Hsf1	Hsf4	Otx2
MP:0002864	abnormal ocular fundus morphology	Crb1	Gpr143	Mitf	Pitx3	Rd9	Rp1h	
MP:0002638	abnormal pupillary reflex	Cat4	Cnga3	Cry1	Eccp	Foxe3	Iac	tmgc25
MP:0002699	abnormal vitreous body	Aldh1a1	Aldh1a3	Bmp4	Cdkn2a	Fzd4	Gas1	
MP:0001314	corneal opacity	Alm	Apo	Areg	Bmp4	Cat4	Col4a1	Lim2
MP:0001851	eye inflammation	Adam17	Atf2	Eda	Fign	ITGA2	ITGA5	Dsc1
MP:0005542	corneal vascularization	Dstn	Eda	Fign	Flt1	Foxe3	Ifnar1	Plg
MP:0003011	delayed dark adaptation	Rbp1	Rdh11	Rdh12	Rdh5	Rdh8	Rlbp1	Pgf
MP:0005172	reduced eye pigmentation	Ap3b1	Ap3d1	Hps5	Hps6	Mitf	Nf1	Sema4a

#### Human

Table 4 shows examples of selected genes in each row that have been positively associated with specific disease phenotype keywords. Each human gene symbol is followed by a specific MeSH disease term and the number of times that gene has been positively associated with the term, in declining order. A major feature of Table 4 is that individual genes have been positively associated with sometimes overlapping disease phenotypes over a broad range from more frequently to less frequently. Table 4 is a small representative subset, truncated in the number of genes (rows) and the number of MeSH terms (columns). The complete list of 1,584 human genes with additional information can be found in Table S1a [20]. An interactive version of the same list can be found in Table S1b[21].

Quite often the resulting list of phenotypes associated with a specific gene may include the major disease phenotype followed by specific sub-phenotypes of the disease that contribute distinct aspects to the overall clinical disease phenotype. For example, IL13 has been associated with asthma at least 11 times as well as to the asthma sub-phenotype immediate hypersensitivity 4 times. Similarly, the gene CFH has been associated with macular degeneration at least 19 times, as well as to the endo-phenotype of macular degeneration, choroidal neovascularization 3 times. Although replication in genetic association studies has been widely debated[22], consistent replication by independent groups, although sometimes with both modest risk and significance values[23], suggests a fundamental measure of scientific validity. This is true for both candidate gene as well as GWAS studies.

In other cases, individual genes have been associated with independent but related disorders that may share fundamental biological pathways in disease etiology, such as HLA-DQB1, CTLA4, and PTPN22 as in the case of autoimmune disorders. This gene overlap emphasizes the fundamental, often step-wise biochemical role each gene plays in shared disease etiology [24-27]. That is, HLA-DQB1 in antigen presentation, CTLA4 in regulation of the expansion of T cell subsets, and PTPN22 in T cell receptor signaling, all contributing to immunological aberrations and progression to clinical disease, as in rheumatoid arthritis, systemic lupus erythematosus, and type 1 diabetes. In other cases, the same gene has been associated with quite different clinical phenotypes, suggesting sharing of complex biological mechanisms at a more underlying level. For example, the gene CFTR, widely recognized as the cause of cystic fibrosis, has been consistently associated with pancreatitis, may be implicated in chronic rhinitis [28], and may play a protective role in gastrointestinal disorders [29].

#### Mouse

Tables 5 and S2 are the mouse equivalents of the human GENE-to-Disease/Phenotype lists (tables 4 and S1 for human). These were developed from the mouse phenotype table of genes with mouse phenotype ontological codes ftp://ftp.informatics.jax.org/pub/reports/index.html#pheno, downloaded on 4-4-08. To build tables 5 and S2, the matching phenotypic terms were exchanged for each Mammalian Phenotype code (MP:#). This resulted in the mouse GENE-to-Disease/Phenotype tables (tables 5 and S2) similar in structure to human GENE-to-Disease/Phenotype tables (tables 4 and S1). Unlike the human tables, the mouse GENE-to-Disease/Phenotype tables come from individual mouse experimental knockout or other genetic studies. They are not based on population based epidemiological studies. They also do not have the quantitative aspect of the human tables with publication frequency counts tagged to each record. In addition, although they include a wide variety of physiological, neurological, and behavioral phenotypes, they do emphasize developmental studies and observational morphological phenotypes common in mouse knockout studies. Table 5 is a small representative subset, truncated in the number of genes (rows) and the number of Phenotype terms (columns). The complete list of 5011 mouse genes with annotated phenotypes and additional information can be found in Table S2a[30]. An interactive version of the same list can be found in Table S2b[31].

#### Direct comparison of human and mouse genes disease/phenotypes

We can now compare these tables directly, thereby allowing gene-by-gene comparison of human disease phenotypes and mouse genetic phenotypes. Tables 6 and S3 are comparisons of the genes that overlap between the human and mouse gene lists (Table S1 and Table S2) showing mouse gene symbols and their human orthologs. Table 6 is a small subset of selected gene-phenotype cross species comparisons. Even though in some cases the human studies have not been replicated, there is often a striking concordance between human disease phenotypes and mouse genetically determined phenotypes. For example, the human gene inhibin alpha (INHA) has been associated with premature ovarian failure[32], and shows mouse phenotypes of abnormal ovarian follicle morphology, female infertility, and ovarian hemorrhage[33], among other phenotypes relevant to human disease. Similarly, in humans the engrailed homeobox 2 gene (EN2) has been associated with autistic disorder[34] while the comparison to mouse En2 has genetic mutations involved in abnormal social integration, spatial learning, and social/consecutive interaction, among others[35]. Importantly, the few mouse studies highlighted above, and many found in the main table S3, were published *after *the corresponding human genetic population based epidemiological studies. Given concerns of false positives and publication bias in human genetic association studies, direct comparisons to related mouse phenotypes may provide supporting evidence that a given gene may be relevant to a specific human disease phenotype. Table S3[36] is a full listing of the 1104 shared genes between the human disease and mouse phenotype summaries.

### Summaries of phenotypes and genes in human and mouse

The second type of main summary tables are DISEASE/PHENOTYPE-to-Gene lists. Disease/Phenotype gene summaries are essentially transposed versions of the GENE-to-Disease/Phenotype summaries (Tables S1 & S2) that allow different types of comparisons. These are non-redundant lists of phenotype keywords, MeSH disease terms in the case of human and Mammalian Phenotype Terms (MP) in the case of mouse, followed by the genes associated or annotated to those disease phenotype keywords.

#### Human

Table 7 shows examples of selected human disease phenotypes in each row positively associated with specific human genes for 8 major MeSH disease classes including cardiovascular, digestive system diseases, diseases of environmental origin, immune system diseases, mental disorders, nervous system diseases, nutritional and metabolic diseases, and eye diseases. Each Mesh phenotype term is followed by the number of times that a specific disease term has been positively associated with a particular gene in each row, in decreasing order. Table 7 is a small representative set, truncated in the number of disease phenotypes (rows) and the number of genes (columns). The complete list of 1,318 MeSH disease phenotype terms with additional information can be found in Table S4a[37]. An interactive version of the complete list can be found in Table S4b[38].

#### Mouse

Tables 8 and S5 constitute the mouse DISEASE/PHENOTYPE-to-Gene summaries. Table 8 consists of selected mouse phenotypes which fall into similar general classes of the human table 7 followed by 6 representative genes that have been assigned to the appropriate phenotypic term due to a specific mouse genetic model. Unlike the human Disease/Phenotype-to-gene tables 7 and S4, the mouse tables 8 and S5 do not have quantitative information. Table 8 is also a small representative set, truncated in the number of disease phenotypes (rows) and the number of genes (columns). The complete list of 5,142 mouse phenotype terms with their corresponding Mammalian PhenoCode designations can be found in Table S5a[39]. An interactive version of the complete list can be found in Table S5b[40].

### Using disease and gene lists

The purpose of this project is not simply to generate lists and information. It is to provide a distillation of disease and phenotype information that can be used in dissecting the complexities of human disease and mouse biology. Now that we have generated GENE-to-disease/phenotype summaries and DISEASE/PHENOTYPE-to-gene summaries for both mouse and human, they can be used for systematic analysis, comparison, and integrating of orthologous data with the goal of providing higher order interpretations of human disease and mouse genetically determined phenotypes.

#### Human disease and mouse phenotype based gene sets

Gene sets have been defined simply as groups of genes that share common biological function, chromosomal location, or regulation[41]. Gene sets are used in high-throughput systematic analysis of microarray data using a priori knowledge. Unlike previously defined gene sets based on biological pathways or differentially expressed genes[41], GAD disease gene sets are unique in that they are composed of genes that have been previously shown to be both polymorphic and have been determined to be genetically positively associated with a specific disease phenotype in a human population based genetic association study. Similarly, Table S5a[39] the mouse DISEASE/PHENOTYPE-to-Gene list is used as a source for gene sets for mouse phenotypes (MP gene sets) comprised of unique gene based mouse genetic models. These gene set files are currently the largest set of gene set files publicly available and the only gene sets files where each gene is based on direct human or mouse genetic studies.

#### Comparison of individual GAD disease gene sets

**Figure 1:**
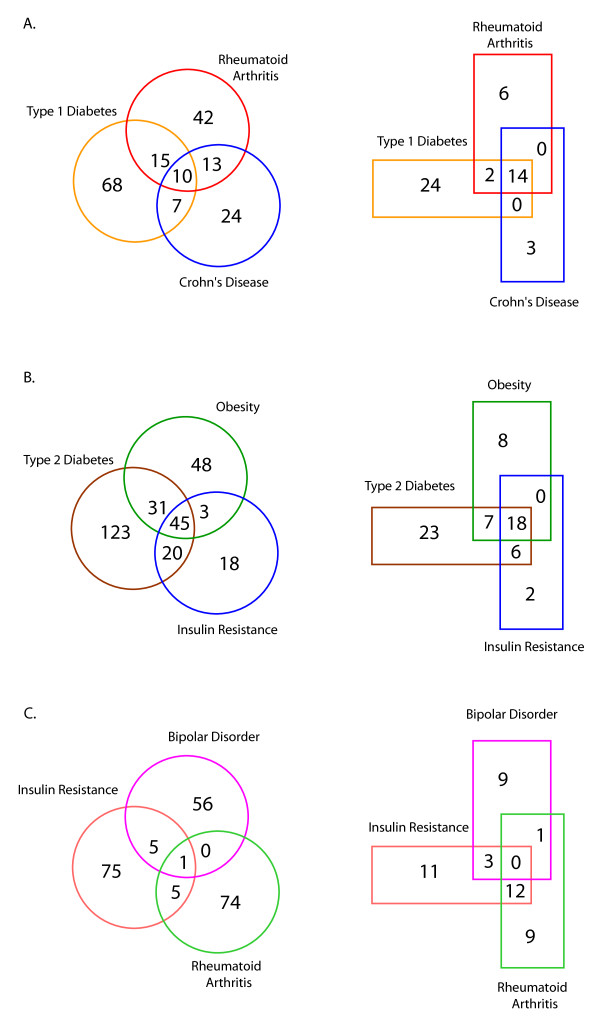
**Venn Diagram analysis of individual GAD disease gene sets (circles) versus pathways (rectangles) produced from the corresponding gene set**. All Venn Diagrams were produced with Venny http://bioinfogp.cnb.csic.es/tools/venny/index.html.

One aspect of common complex disease is that the development of disease and disease phenotypes quite often present along a broad spectrum of symptoms and share clinical characteristics, endo-phenotypes, or quantitative traits with closely related disorders [25]. This is evident in gene sharing, as mentioned above, and equally in the overlap of biological pathways between related disorders. Using GAD disease gene sets, Venn diagram comparisons among related disorders shows modest gene sharing. However, when gene sets are then placed into biological pathways and compared by Venn analysis, there is a marked increase in the overlap in pathways between related disorders. This was not found in gene sets from unrelated disorders. For example, major autoimmune disorders quite often share endophenotypes of lymphoproliferation, autoantibody production, and alterations in apoptosis, as well as other immune cellular and biochemical aberrations. As shown in Figure 1a, genes that have been positively associated with type 1 diabetes, rheumatoid arthritis, and Crohn's disease show a modest overlap. However, when individual gene sets are fitted into biological pathways, then compared for overlap of pathway membership, there is a striking increase in the overlap at the pathway level. This is true in a comparison of gene and pathways for type 2 diabetes, insulin resistance, and obesity as well (Figure 1b). This pattern of major pathway overlap does not seem to occur between unrelated disorders, such as insulin resistance, rheumatoid arthritis and bipolar disorder (Figure 1c). This disease related sharing at the pathway level suggests common regulatory mechanisms between these disorders and that the original positive associations are not necessarily due to random chance alone.

#### Group analysis of GAD disease gene sets between major classes of disease/phenotypes

##### Dendrogram analysis of human disease gene sets

**Figure 2:**
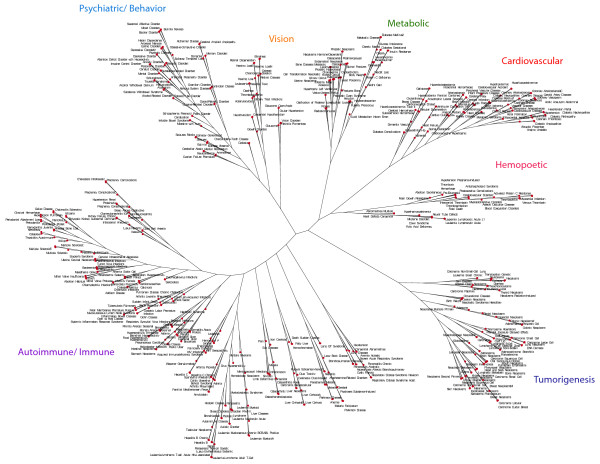
**Human dendrogram comparison of 480 GAD disease gene sets based on gene sharing**. The input GAD gene set file for this figure can be found in Table S6[46].

As archival information grows, analysis of complex molecular and genetic datasets using clustering or network approaches has become increasingly more useful [13,42-45]. Therefore, in addition to comparisons between individual diseases using human and mouse gene sets, we analyzed large gene groups using dendrogram and clustering approaches based on gene sharing between gene sets. Figure 2 shows a broad based dendrogram comparison based on gene sharing between 480 GAD disease gene sets, using gene sets each containing at least 3 genes. A striking feature of this analysis is that at a coarse level, major disease groups cluster together in space demonstrating shared genes between major clinically important disease groups. Disease domains are represented by groups such as cardiovascular disorders, metabolic disorders, cancer, immune and inflammatory disorders, vision, and chemical dependency. At finer detail within a specific broader group, it becomes clear that individual diseases with overlapping phenotypes are found close in space, such as asthma, allergic rhinitis, and atopic dermatitis. This overlap due to gene sharing recapitulates an overlap in clinical characteristics between these related disorders. Similarly, phenotypes within the metabolic group related to diabetes are closely aligned in space including; insulin resistance, hyperglycemia, hyperinsulinemia, and hyperlipidemia. This close apposition of related disease phenotypes and sub-phenotypes at both a coarse and fine level is a consistent feature of the overall display. The human gene sets used in creating this tree diagram can be found in Table S6[46]. It is important to emphasize that this display and the distance relationships between diseases are calculated through an unbiased gene-sharing algorithm independent of disease phenotype labels and not as a result of an imposed logical hierarchy or an ontological annotation system. This grouping of major disease phenotypes based solely on gene sharing provides supporting evidence that the underlying disease based gene sets may have a fundamental relevance to disease and may not be reported in the literature by chance alone.

##### Dendrogram analysis of mouse phenotypic gene sets

**Figure 3:**
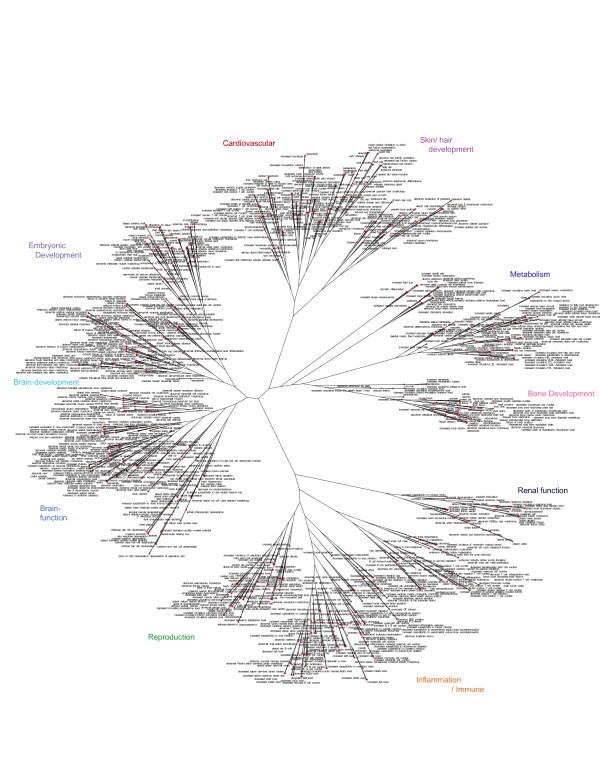
**Mouse dendrogram comparison of 1056 mouse phenotype (MP) gene sets based on gene sharing**. The input MP gene set file for this figure can be found in Table S7[47].

Figure 3 is a similar dendrogram to the human tree using 1056 mouse phenotypic gene sets, using gene sets each containing at least 10 genes. This was produced using the same gene sharing algorithm as for the human gene sets in Figure 2. As with the human dendrogram, the mouse tree displays informative groupings at both a coarse and fine level. This tree groups into major groupings nominally assigned as brain development and brain function, embryonic development, cardiovascular, reproduction, inflammation, renal function, bone development, metabolism, and skin/hair development. The identification of major groupings emphasizing developmental processes reflects the emphasis of gene knockouts and developmental models resulting in observable morphological traits and less so with regard to end stage clinical diseases as in the human dendrogram. Like the human dendrogram (Figure 2) discrete major functional groupings in the mouse dendrogram suggests that individual experimental observations are not random. Fundamental complex processes such as metabolism, cardiovascular phenomena, and developmental processes are integrated by extensive sharing of related pliotropic genes. Moreover, like the human tree, fine structure in the mouse tree shows related mouse phenotypes are closely positioned in space. For example, in the metabolism major grouping, the individual phenotypes of body mass, adipose phenotypes, and weight gain are closely positioned. Similarly, in the brain function group, the behavioral phenotypes of anxiety, exploration, and responses to novel objects are found next to one another. This pattern is a fundamental feature of this tree. Like the human tree, the mouse dendrogram shown here is based solely on a gene sharing algorithm using genes assigned to individual phenotypes. It is not based on an imposed predetermined hierarchy or ontology. Importantly, unlike the human tree, the information contained in the mouse tree is derived from individual independent mouse genetic studies and phenotypic observations and not from large case controlled population based epidemiological studies. Controversial issues such as publication bias or study size which confound human genetic association studies are not as relevant here in the context of studies of experimentally determined individual mouse gene knockouts and related studies. The mouse gene sets used in creating this tree diagram can be found in Table S7[47].

##### Hierarchical clustering of human and mouse gene sets

Hierarchical clustering has become a common tool in the analysis of large molecular data sets[48] allowing identification of similar patterns in a scalable fashion from the whole experiment down to a level of fine structure. To provide further evidence of disease relevance and biological content contained in both the human and mouse gene sets hierarchical clustering was performed on both human and mouse. Four hundred and eighty human gene sets were clustered producing 46 major disease clusters. In the mouse, clustering was performed on 2067 mouse phenotype gene sets, using gene sets containing at least 3 genes. This resulted in 165 major subgroups of functional phenotypic specificity. Hierarchical clustering is shown for human [Additional file 1 and Additional file 2] and for mouse [Additional file 3 and Additional file 4]. Like the human and mouse dendograms, this hierarchical clustering showed functional disease grouping at both a coarse group level and at a fine level within major phenotypic groupings. These clusters in both human and mouse falling into closely defined broad functional groups as well as closely related clinical, physiological, and developmental phenotypes demonstrates a general pattern of relevance to disease in their original underlying genetic associations. As in the dendrogram displays, this suggests that the genes nominally positively associated to these disorders, drawn from the medical literature, are not pervasively randomly assigned or due to a widespread pattern of random false positives associations.

## Discussion and Conclusion

This report describes a summary of the positive genetic associations to disease phenotypes found in the Genetic Association Database as well as a summary of mouse genetically determined phenotypes from the MGI phenotypes database. The genes and disease lists described here were derived from a broad literature mining approach. We have shown disease relevance in three distinct ways; a) in comparing individual gene lists and pathways, b) comparing between species and, c) in broad based comparative analysis utilizing complex systems approaches. Moreover, we identify disease based genes sets for 1,317 human disease phenotypes as well as 5,142 mouse experimentally determined phenotypes. These resources are the largest gene set files currently publicly available and the only gene set files derived from population based human epidemiological genetic studies and mouse genetic models of disease.

Each individual GAD disease gene set (i.e. a single disease term followed by a string of genes) or mouse phenotype gene set becomes a candidate for a number of uses and applications including:

a) contributing to complex (additive, multiplicative, gene-environment) statistical models for any given disease phenotype [49-53]; b) use in comparative analysis of disease between disease phenotypes; c) use in interrogating other related data types, such as microarray (see below), proteomic, or SNP data [54-56]; and d) integration into annotation engines[57] or genome browsers[58] or other analytical software to add disease information in comparative genomic analysis. In a sense, each individual human or mouse disease/phenotype gene set becomes a unique hypothesis, testable in a variety of ways. Increasingly, combinations of genes may have important predictive value as combinatorial biomarkers in predicting disease risk as opposed to single candidate genes [59,60].

In addition, in an ongoing parallel set of experiments, using a Gene Set Analysis (GSA) approach using the web tool Disease/Phenotype web-PAGE, in the analysis of orthologous microarray data (De S, Zhang Y, Garner JR, Wang SA, Becker KG: Disease and phenotype gene set analysis of disease based gene expression, unpublished), both the human and mouse disease/phenotype gene sets defined above demonstrate striking disease specificity in PAGE[61] gene set analysis of previously published microarray based gene expression studies from numerous independent laboratories in both a species specific and cross species manner. This was true when studying gene expression studies of type 2 diabetes, obesity, myocardial infarction and sepsis, among others, providing further evidence of the disease and clinical relevance of both the human and mouse gene sets.

This approach is limited in a number of ways. In particular, the GAD database compares the results of human population based epidemiological studies performed using different sample sizes, populations, statistical models, and at different times over approximately the last 16 years. In addition, the GAD database draws on association studies of broad quality with different degrees of detail provided. Although all human genetic association studies discussed here have been individually determined to be positively associated with a disease or phenotype in a peer reviewed journal, we make no assertion that any individual study is correct and we recognize the controversy in the genetics community regarding statistical and biological significance of genetic association studies. Moreover, although the GAD database contains information on polymorphism and variation, and each GAD record is fundamentally based on polymorphism, this report does not consider variation or polymorphism in the summaries shown. Likewise, mouse genetic models in many cases are weighted to gene knockouts which may not be necessarily be directly representative of multifactorial human common complex disease.

However, even with these limitations, we believe valuable insights can be gained from broad based literature assessments of the genetic contribution in human common complex disease and in mouse phenotypic biology. More importantly, this suggests greater opportunities for systematic mining and analysis of published data and in cross comparison of archival molecular databases in both human and animal models of disease with regard to genetic variation, population comparisons, and integration with many different types of orthologous information.

## Abbreviations

GAD: Genetic Association Database; MGI: Mouse Genome Informatics; MeSH: Medical Subject Headings; GWAS: Genome Wide Association Study; CDC: Centers for Disease Control and Prevention; HuGENet: Human Genome Epidemiology Network.

## Competing interests

The authors declare that they have no competing interests.

## Authors' contributions

YZ performed statistical analysis, gene set assembly, and contributed to the manuscript. SD performed dendrogram and clustering analysis and contributed to the manuscript. JG, KS, and SAW did database curation and analysis. KGB organized the project, did database curation, performed comparisons, and wrote the manuscript. All authors read and approved the manuscript.

## Pre-publication history

The pre-publication history for this paper can be accessed here:

http://www.biomedcentral.com/1755-8794/3/1/prepub

## Supplementary Material

Additional file 1 **Hierarchical clustering of 480 Human GAD disease gene sets**. This file contains a display of hierarchical clustering of 480 Human GAD disease gene sets, each gene set contain at least 3 genes each. Click here for file

Additional file 2 **Individual human disease functional clusters**. This file contains selected subsets of Additional File 1 including; a. tumorigenesis, b. autoimmune, c. cardiovascular, d. metabolism, and e. behavior. Click here for file

Additional file 3 **Hierarchical clustering of 2067 Mouse phenotypic gene sets**. This file contains a display of hierarchical clustering of 2067 Mouse phenotypic gene sets, each gene set contain at least 10 genes each. Click here for file

Additional file 4 **Individual mouse phenotypic functional clusters**. This file contains selected subsets of Additional File 2 including; a. immune function, b. metabolism, c. neurological function/behavior, d. DNA replication/tumorigenesis, e. development and f. cardiovascular. Click here for file

## References

[B1] Eyre TA Ducluzeau F Sneddon TP Povey S Bruford EA Lush MJ The HUGO Gene Nomenclature Database, 2006 updates Nucleic Acids Res 2006 34 Database D319 321 10.1093/nar/gkj147 16381876 PMC1347509

[B2] Becker KG Barnes KC Bright TJ Wang SA The genetic association database Nat Genet 2004 36 5 431 432 10.1038/ng0504-431 15118671

[B3] Lin BK Clyne M Walsh M Gomez O Yu W Gwinn M Khoury MJ Tracking the epidemiology of human genes in the literature: the HuGE Published Literature database Am J Epidemiol 2006 164 1 1 4 10.1093/aje/kwj175 16641305

[B4] Bult CJ Eppig JT Kadin JA Richardson JE Blake JA The Mouse Genome Database (MGD): mouse biology and model systems Nucleic Acids Res 2008 36 Database D724 728 18158299 10.1093/nar/gkm961 PMC2238849

[B5] Hancock JM Adams NC Aidinis V Blake A Bogue M Brown SD Chesler EJ Davidson D Duran C Eppig JT Mouse Phenotype Database Integration Consortium: integration [corrected] of mouse phenome data resources Mamm Genome 2007 18 3 157 163 10.1007/s00335-007-9004-x 17436037 PMC4230762

[B6] McKusick VA Mendelian Inheritance in Man and its online version, OMIM Am J Hum Genet 2007 80 4 588 604 10.1086/514346 17357067 PMC1852721

[B7] Adie EA Adams RR Evans KL Porteous DJ Pickard BS SUSPECTS: enabling fast and effective prioritization of positional candidates Bioinformatics 2006 22 6 773 774 10.1093/bioinformatics/btk031 16423925

[B8] Yue P Melamud E Moult J SNPs3D: candidate gene and SNP selection for association studies BMC Bioinformatics 2006 7 166 10.1186/1471-2105-7-166 16551372 PMC1435944

[B9] Smink LJ Helton EM Healy BC Cavnor CC Lam AC Flamez D Burren OS Wang Y Dolman GE Burdick DB T1DBase, a community web-based resource for type 1 diabetes research Nucleic Acids Res 2005 33 Database D544 549 15608258 10.1093/nar/gki095 PMC540049

[B10] Sherman BT Huang DW Tan Q Guo Y Bour S Liu D Stephens R Baseler MW Lane HC Lempicki RA DAVID Knowledgebase: a gene-centered database integrating heterogeneous gene annotation resources to facilitate high-throughput gene functional analysis BMC Bioinformatics 2007 8 1 426 10.1186/1471-2105-8-426 17980028 PMC2186358

[B11] Yi M Horton JD Cohen JC Hobbs HH Stephens RM WholePathwayScope: a comprehensive pathway-based analysis tool for high-throughput data BMC Bioinformatics 2006 7 30 10.1186/1471-2105-7-30 16423281 PMC1388242

[B12] Jegga AG Chen J Gowrisankar S Deshmukh MA Gudivada R Kong S Kaimal V Aronow BJ GenomeTrafac: a whole genome resource for the detection of transcription factor binding site clusters associated with conventional and microRNA encoding genes conserved between mouse and human gene orthologs Nucleic Acids Res 2007 35 Database D116 121 10.1093/nar/gkl1011 17178752 PMC1781107

[B13] Butte AJ Kohane IS Creation and implications of a phenome-genome network Nat Biotechnol 2006 24 1 55 62 10.1038/nbt1150 16404398 PMC2716377

[B14] VENNY. An interactive tool for comparing lists with Venn Diagrams http://bioinfogp.cnb.csic.es/tools/venny/index.html

[B15] Zhang B Kirov S Snoddy J WebGestalt: an integrated system for exploring gene sets in various biological contexts Nucleic Acids Res 2005 33 Web Server W741 748 10.1093/nar/gki475 15980575 PMC1160236

[B16] PHYLIP http://evolution.gs.washington.edu/phylip.html

[B17] Fitch WM Margoliash E Construction of phylogenetic trees Science 1967 155 760 279 284 10.1126/science.155.3760.279 5334057

[B18] Choi JH Jung HY Kim HS Cho HG PhyloDraw: a phylogenetic tree drawing system Bioinformatics 2000 16 11 1056 1058 10.1093/bioinformatics/16.11.1056 11159323

[B19] Ward J Hierarchical Grouping to optimize an objective function Journal of American Statistical Association 1963 58 301 236 244 10.2307/2282967

[B20] Table S1a-Human GENE-to-Disease/Phenotype. A file of Human Genes followed by Disease Phenotype MeSH terms http://www.grc.nia.nih.gov/branches/rrb/dna/data/table-s1-a.txt

[B21] Table S1b-Human GENE-to-Disease/Phenotype interactive The same list as Table S1a, but with direct searches back to GAD http://www.grc.nia.nih.gov/branches/rrb/dna/data/table-s1-b.html

[B22] Ioannidis JP Why most published research findings are false PLoS Med 2005 2 8 e124 10.1371/journal.pmed.0020124 16060722 PMC1182327

[B23] Khoury MJ Little J Gwinn M Ioannidis JP On the synthesis and interpretation of consistent but weak gene-disease associations in the era of genome-wide association studies Int J Epidemiol 2007 36 2 439 445 10.1093/ije/dyl253 17182636

[B24] Becker KG Simon RM Bailey-Wilson JE Freidlin B Biddison WE McFarland HF Trent JM Clustering of non-major histocompatibility complex susceptibility candidate loci in human autoimmune diseases Proc Natl Acad Sci USA 1998 95 17 9979 9984 10.1073/pnas.95.17.9979 9707586 PMC21447

[B25] Becker KG The common variants/multiple disease hypothesis of common complex genetic disorders Med Hypotheses 2004 62 2 309 317 10.1016/S0306-9877(03)00332-3 14962646

[B26] Lee YH Rho YH Choi SJ Ji JD Song GG Nath SK Harley JB The PTPN22 C1858T functional polymorphism and autoimmune diseases--a meta-analysis Rheumatology (Oxford) 2007 46 1 49 56 10.1093/rheumatology/kel170 16760194

[B27] Plenge RM Padyukov L Remmers EF Purcell S Lee AT Karlson EW Wolfe F Kastner DL Alfredsson L Altshuler D Replication of putative candidate-gene associations with rheumatoid arthritis in >4,000 samples from North America and Sweden: association of susceptibility with PTPN22, CTLA4, and PADI4 Am J Hum Genet 2005 77 6 1044 1060 10.1086/498651 16380915 PMC1285162

[B28] Wang X Kim J McWilliams R Cutting GR Increased prevalence of chronic rhinosinusitis in carriers of a cystic fibrosis mutation Arch Otolaryngol Head Neck Surg 2005 131 3 237 240 10.1001/archotol.131.3.237 15781764

[B29] Bresso F Askling J Astegiano M Demarchi B Sapone N Rizzetto M Gionchetti P Lammers KM de Leone A Riegler G Potential role for the common cystic fibrosis DeltaF508 mutation in Crohn's disease Inflamm Bowel Dis 2007 13 5 531 536 10.1002/ibd.20067 17206681

[B30] Table S2a-Mouse GENE-to-Disease/Phenotype. A file of Mouse Genes followed by Disease Phenotype Mammalian Phenotype (MP) terms http://www.grc.nia.nih.gov/branches/rrb/dna/data/table-s2-a.txt

[B31] Table S2b-Mouse GENE-to-Disease/Phenotype interactive. The same list as Table S2a, but with direct searches back to MGI and GAD http://www.grc.nia.nih.gov/branches/rrb/dna/data/table-s2-b.html

[B32] Harris SE Chand AL Winship IM Gersak K Nishi Y Yanase T Nawata H Shelling AN INHA promoter polymorphisms are associated with premature ovarian failure Mol Hum Reprod 2005 11 11 779 784 10.1093/molehr/gah219 16390856

[B33] Wu X Chen L Brown CA Yan C Matzuk MM Interrelationship of growth differentiation factor 9 and inhibin in early folliculogenesis and ovarian tumorigenesis in mice Mol Endocrinol 2004 18 6 1509 1519 10.1210/me.2003-0399 15016837

[B34] Gharani N Benayed R Mancuso V Brzustowicz LM Millonig JH Association of the homeobox transcription factor, ENGRAILED 2, 3, with autism spectrum disorder Mol Psychiatry 2004 9 5 474 484 10.1038/sj.mp.4001498 15024396

[B35] Cheh MA Millonig JH Roselli LM Ming X Jacobsen E Kamdar S Wagner GC En2 knockout mice display neurobehavioral and neurochemical alterations relevant to autism spectrum disorder Brain Res 2006 1116 1 166 176 10.1016/j.brainres.2006.07.086 16935268

[B36] Table S3-Human-Mouse Gene Overlap. A list of 1105 genes that overlap between the Human GENE-to-Disease Phenotype list (S1) and the Mouse GENE-to-Disease phenotype list (S2) http://www.grc.nia.nih.gov/branches/rrb/dna/data/table-s3.txt

[B37] Table S4a-Human DISEASE/PHENOTYPE-to-Gene. A file of Human Disease Phenotype MeSH terms followed by associated genes http://www.grc.nia.nih.gov/branches/rrb/dna/data/table-s4-a.txt

[B38] Table S4b-Human DISEASE/PHENOTYPE-to-Gene Interactive. A file of Human Disease Phenotype MeSH terms followed by associated genes, but with direct searches back to GAD http://www.grc.nia.nih.gov/branches/rrb/dna/data/table-s4-b.html

[B39] Table S5a-Mouse DISEASE/PHENOTYPE-to-Gene (mouse). A file of Mouse Disease-Phenotype Mammalian Phenotype (MP) terms followed by assigned mouse genes http://www.grc.nia.nih.gov/branches/rrb/dna/data/table-s5-a.txt

[B40] Table S5b-Mouse DISEASE/PHENOTYPE-to-Gene (mouse) Interactive. A file of Mouse Disease-Phenotype Mammalian Phenotype (MP) terms followed by assigned mouse genes, but with direct searches back to MGI http://www.grc.nia.nih.gov/branches/rrb/dna/data/table-s5-b.html

[B41] Subramanian A Tamayo P Mootha VK Mukherjee S Ebert BL Gillette MA Paulovich A Pomeroy SL Golub TR Lander ES Gene set enrichment analysis: a knowledge-based approach for interpreting genome-wide expression profiles Proc Natl Acad Sci USA 2005 102 43 15545 15550 10.1073/pnas.0506580102 16199517 PMC1239896

[B42] Liu M Liberzon A Kong SW Lai WR Park PJ Kohane IS Kasif S Network-based analysis of affected biological processes in type 2 diabetes models PLoS Genet 2007 3 6 e96 10.1371/journal.pgen.0030096 17571924 PMC1904360

[B43] Goh KI Cusick ME Valle D Childs B Vidal M Barabasi AL The human disease network Proc Natl Acad Sci USA 2007 104 21 8685 8690 10.1073/pnas.0701361104 17502601 PMC1885563

[B44] Emilsson V Thorleifsson G Zhang B Leonardson AS Zink F Zhu J Carlson S Helgason A Walters GB Gunnarsdottir S Genetics of gene expression and its effect on disease Nature 2008 452 7186 423 428 10.1038/nature06758 18344981

[B45] Guan Y Myers CL Lu R Lemischka IR Bult CJ Troyanskaya OG A genomewide functional network for the laboratory mouse PLoS Comput Biol 2008 4 9 e1000165 10.1371/journal.pcbi.1000165 18818725 PMC2527685

[B46] Table S6-Human Dendrogram Gene Sets. A file of the GAD Human gene sets used in the dendrogram fig 2 http://www.grc.nia.nih.gov/branches/rrb/dna/data/table-s6.txt

[B47] Table S7-Mouse Dendrogram Gene Sets. A file of the Mouse gene sets used to build the mouse dendrogram fig 3 http://www.grc.nia.nih.gov/branches/rrb/dna/data/table-s7.txt

[B48] Eisen MB Spellman PT Brown PO Botstein D Cluster analysis and display of genome-wide expression patterns Proc Natl Acad Sci USA 1998 95 25 14863 14868 10.1073/pnas.95.25.14863 9843981 PMC24541

[B49] Evans DM Visscher PM Wray NR Harnessing the information contained within genome-wide association studies to improve individual prediction of complex disease risk Hum Mol Genet 2009 18 18 3525 3531 10.1093/hmg/ddp295 19553258

[B50] Wray NR Goddard ME Visscher PM Prediction of individual genetic risk of complex disease Curr Opin Genet Dev 2008 18 3 257 263 10.1016/j.gde.2008.07.006 18682292

[B51] Heidema AG Boer JM Nagelkerke N Mariman EC van der AD Feskens EJ The challenge for genetic epidemiologists: how to analyze large numbers of SNPs in relation to complex diseases BMC Genet 2006 7 23 10.1186/1471-2156-7-23 16630340 PMC1479365

[B52] Mei H Cuccaro ML Martin ER Multifactor dimensionality reduction-phenomics: a novel method to capture genetic heterogeneity with use of phenotypic variables Am J Hum Genet 2007 81 6 1251 1261 10.1086/522307 17999363 PMC2276344

[B53] Slatkin M Exchangeable models of complex inherited diseases Genetics 2008 179 4 2253 2261 10.1534/genetics.107.077719 18689899 PMC2516095

[B54] Chasman DI On the utility of gene set methods in genomewide association studies of quantitative traits Genet Epidemiol 2008 32 7 658 668 10.1002/gepi.20334 18481796

[B55] Holden M Deng S Wojnowski L Kulle B GSEA-SNP: applying gene set enrichment analysis to SNP data from genome-wide association studies Bioinformatics 2008 24 23 2784 2785 10.1093/bioinformatics/btn516 18854360

[B56] Chai HS Sicotte H Bailey KR Turner ST Asmann YW Kocher JP GLOSSI: a method to assess the association of genetic loci-sets with complex diseases BMC Bioinformatics 2009 10 1 102 10.1186/1471-2105-10-102 19344520 PMC2678095

[B57] Huang da W Sherman BT Zheng X Yang J Imamichi T Stephens R Lempicki RA Extracting biological meaning from large gene lists with DAVID Curr Protoc Bioinformatics 2009 Chapter 13 Unit 13 11 19728287 10.1002/0471250953.bi1311s27

[B58] Kuhn RM Karolchik D Zweig AS Wang T Smith KE Rosenbloom KR Rhead B Raney BJ Pohl A Pheasant M The UCSC Genome Browser Database: update 2009 Nucleic Acids Res 2009 37 Database D755 761 10.1093/nar/gkn875 18996895 PMC2686463

[B59] Ray S Britschgi M Herbert C Takeda-Uchimura Y Boxer A Blennow K Friedman LF Galasko DR Jutel M Karydas A Classification and prediction of clinical Alzheimer's diagnosis based on plasma signaling proteins Nat Med 2007 13 11 1359 1362 10.1038/nm1653 17934472

[B60] Zheng SL Sun J Wiklund F Smith S Stattin P Li G Adami HO Hsu FC Zhu Y Balter K Cumulative Association of Five Genetic Variants with Prostate Cancer N Engl J Med 2008 358 9 910 9 10.1056/NEJMoa075819 18199855

[B61] Kim SY Volsky DJ PAGE: parametric analysis of gene set enrichment BMC Bioinformatics 2005 6 144 10.1186/1471-2105-6-144 15941488 PMC1183189

