# Supplementary figures and images for: Systematic analysis, comparison, and integration of disease based human genetic association data and mouse genetic phenotypic information
Source: BMC Med Genomics. 2010 Jan 21;3:1. doi: 10.1186/1755-8794-3-1 (PMC2822734; doi:10.1186/1755-8794-3-1)

## Human GAD Disease Hierarchical Cluster

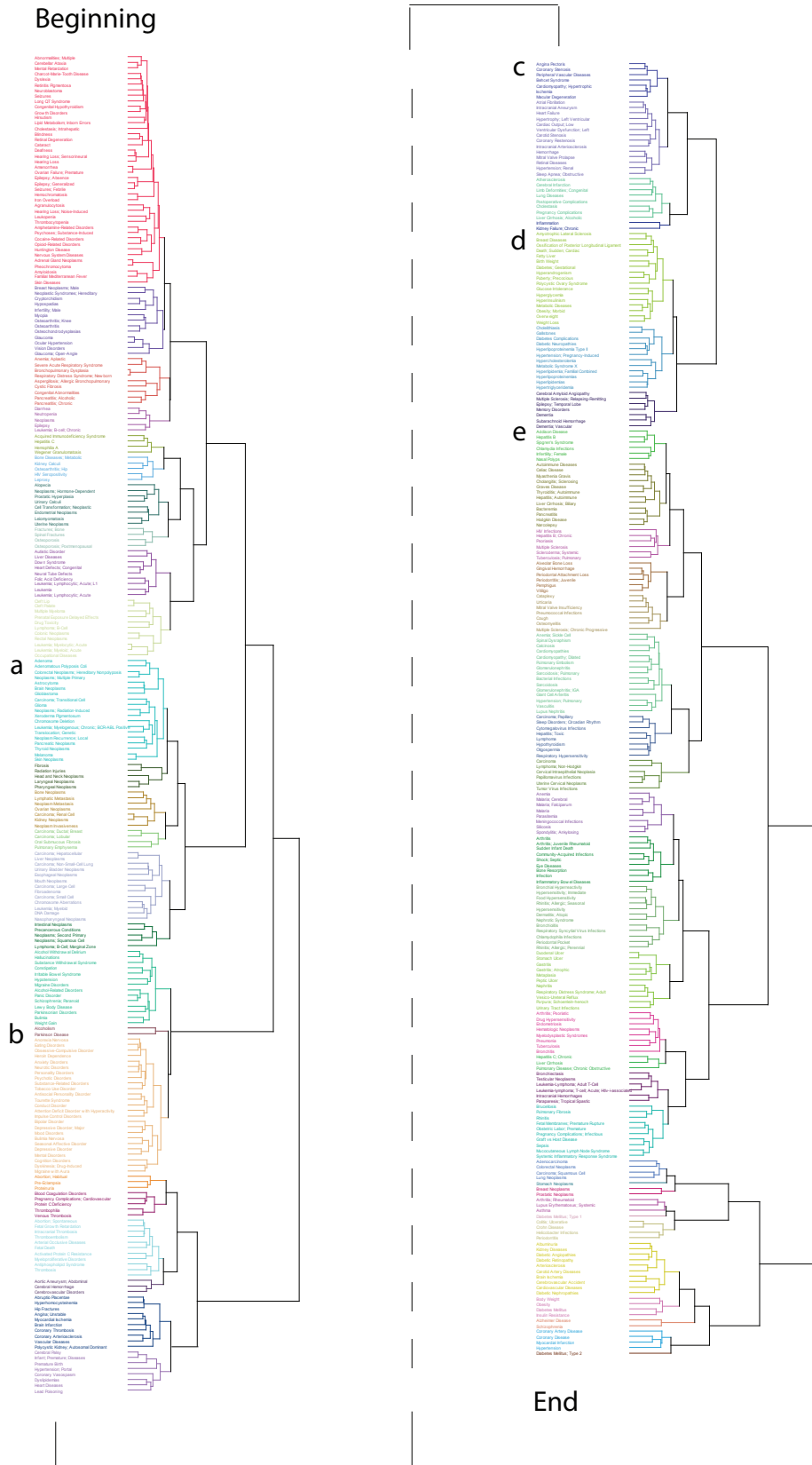

Supplement: Additional file 1 — Hierarchical clustering of 480 Human GAD disease gene sets. This file contains a display of hierarchical clustering of 480 Human GAD disease gene sets, each gene set contain at least 3 genes each. [file 1755-8794-3-1-S1.PDF]

# Mouse Heirarchical Cluster

Beginning

a

b

c

d

e

f

End

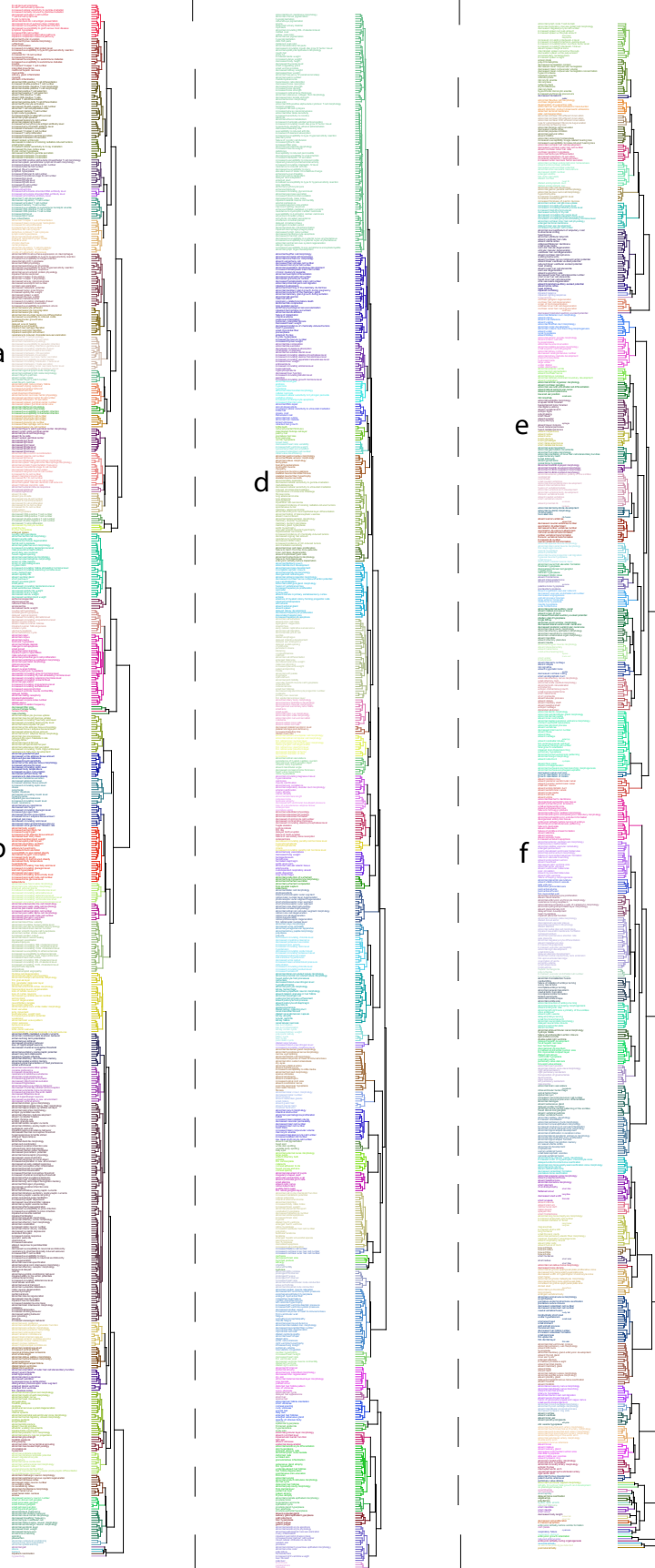

Supplement: Additional file 3 — Hierarchical clustering of 2067 Mouse phenotypic gene sets. This file contains a display of hierarchical clustering of 2067 Mouse phenotypic gene sets, each gene set contain at least 10 genes each. [file 1755-8794-3-1-S3.PDF]
