# Supplementary material for: Systematic analysis, comparison, and integration of disease based human genetic association data and mouse genetic phenotypic information
Source: BMC Med Genomics. 2010 Jan 21;3:1. doi: 10.1186/1755-8794-3-1 (PMC2822734; doi:10.1186/1755-8794-3-1)

Selected human GAD disease functional clusters

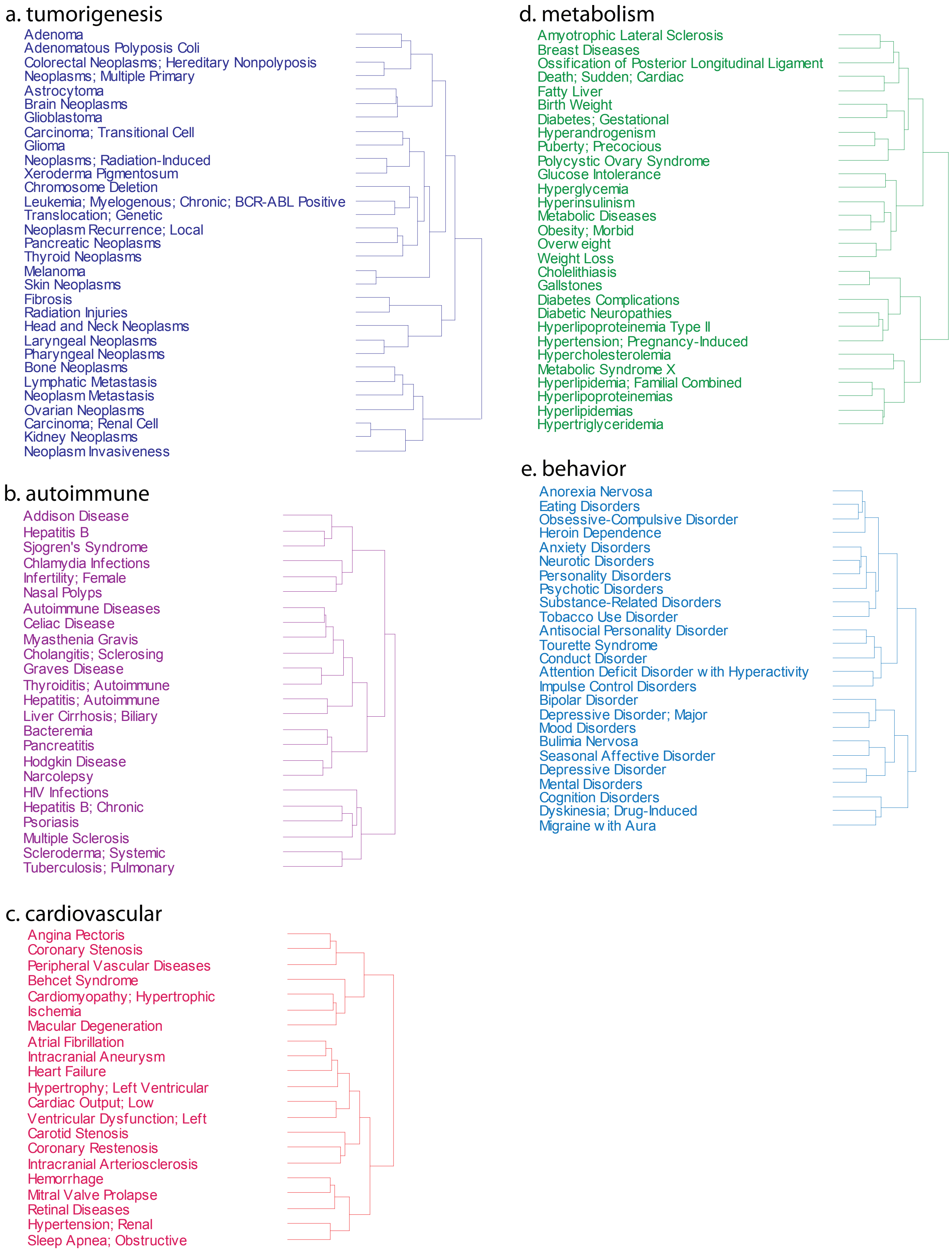

Supplement: Additional file 2 — Individual human disease functional clusters. This file contains selected subsets of Additional File 1 including; a. tumorigenesis, b. autoimmune, c. cardiovascular, d. metabolism, and e. behavior. [file 1755-8794-3-1-S2.PDF]
