# Supplementary material for: Systematic analysis, comparison, and integration of disease based human genetic association data and mouse genetic phenotypic information
Source: BMC Med Genomics. 2010 Jan 21;3:1. doi: 10.1186/1755-8794-3-1 (PMC2822734; doi:10.1186/1755-8794-3-1)

Selected mouse functional clusters

a. immune function

abnormal NK T cell physiology
decreased interleukin-12 secretion
abnormal chemokine physiology
decreased susceptibility to parasitic infection
decreased circulating interferon-gamma level
decreased circulating interleukin-6 level
decreased interferon-alpha secretion
decreased interleukin-12b secretion
decreased interleukin-6 secretion
decreased tumor necrosis factor secretion
increased susceptibility to viral infection
decreased circulating interleukin-1 beta level
decreased interleukin-1 beta secretion
decreased circulating tumor necrosis factor level
decreased susceptibility to endotoxin shock

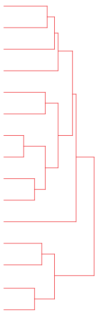

b. metabolism

abnormal body weight
increased percent body fat
increased weight gain
increased white adipose tissue amount
decreased lean body mass
increased seminal gland weight
abnormal epididymal fat pad
abnormal respiratory quotient
increased lean body mass
adipocyte hypertrophy
increased insulin secretion
susceptibility to age related obesity
decreased oxygen consumption
increased body length
susceptibility to diet-induced obesity
decreased body temperature
hyperlipidemia
increased circulating free fatty acid level
increased circulating glucagon level
islet cell hyperplasia
decreased glycogen level
increased circulating ketone body level
increased pancreatic beta cell number
increased urine glucose level
ketoaciduria

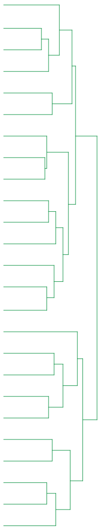

c. neurological function/ behavior

abnormal AMPA-mediated synaptic currents
abnormal conditioned taste aversion behavior
enhanced long term potentiation
abnormal pup retrieval
decreased grooming behavior
loss of hippocampal neurons
decreased chemical nociceptive threshold
jumpy
abnormal excitatory postsynaptic potential
absent long term depression
impaired synaptic plasticity
abnormal olfactory -discrimination memory
abnormal spatial working memory
impaired social transmission of food preference
social withdrawal
abnormal neurotransmitter uptake
cocaine preference
increased dopamine level
resistance to addictive substance
abnormal noradrenaline level
increased aggression to humans
decreased mitochondrial oxidation
increased thigmotaxis
abnormal passive avoidance behavior
decreased chemically-elicited antinociception
abnormal substantia nigra morphology
increased aggression towards males
decreased dopamine level
loss of dopaminergic neurons
decreased exploration in new environment
decreased vertical activity

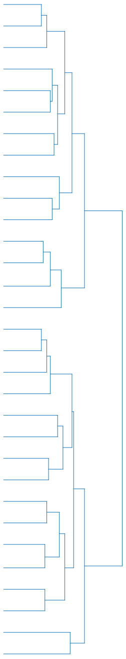

d. DNA replication/ tumorigenesis

abnormal DNA replication
decreased cellular sensitivity to gamma-irradiation
medulloblastoma
decreased cellular sensitivity to ultraviolet irradiation
induced chromosome breakage
spontaneous chromosome breakage
fibrosarcoma
lung adenocarcinoma
lung adenoma
skin papilloma
squamous cell carcinoma
increased incidence of ionizing radiation-induced tumors
spontaneous tumor
mammary adenocarcinoma
abnormal embryonic neuroepithelial layer differentiation
abnormal folding of telencephalic vesicles
absent neurocranium
abnormal metencephalon morphology
mammary alveolar hyperplasia
mammary ductal hyperplasia
mammary gland hyperplasia
aortic hypertrophy
vascular smooth muscle hypertrophy
hepatocellular carcinoma
increased incidence of chemically-induced tumors
decreased zigzag hair amount
resistance to lymphoma
decreased mitotic index
increased incidence of UV-induced tumors
skin irradiation sensitivity

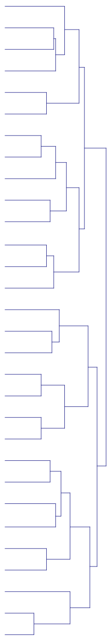

e. development

abnormal epiglottis morphology
scapular bone foramen
basisphenoid bone foramen
interdigital webbing
absent vertebral arch
ectopic digits
polyphalangy
short fibula
absent enamel
polysyndactyly
aphagia
absent lower incisors
fused metacarpal bones
fused metatarsal bones
absent carpal bone
absent radius
absent ulna
brachydactyly
brachyphalangia
short metacarpal bones
short metatarsal bones

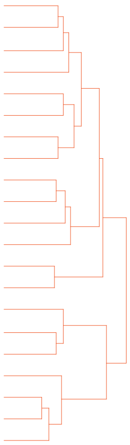

f. cardiovascular

abnormal anterior cardinal vein morphology
arteriovenous malformation
abnormal vitelline vascular remodeling
enlarged pericardium
poorly developed ventricular trabeculae
abnormal endocardium morphology
failure of myocardial trabecular formation
failure of vascular branching
absent endocardial cushion
absent heartbeat
decreased right ventricle size
abnormal sinus venosus
absent ventricular trabeculae
poor circulation
absent myocardial trabeculae
absent right ventricle
failure of looping morphogenesis

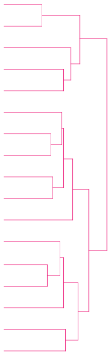

Supplement: Additional file 4 — Individual mouse phenotypic functional clusters. This file contains selected subsets of Additional File 2 including; a. immune function, b. metabolism, c. neurological function/behavior, d. DNA replication/tumorigenesis, e. development and f. cardiovascular. [file 1755-8794-3-1-S4.PDF]
